# Topotactic, Vapor-Phase, *In Situ* Monitored Formation of Ultrathin, Phase-Pure 2D-on-3D Halide Perovskite Surfaces

**DOI:** 10.1021/acsami.3c01881

**Published:** 2023-05-03

**Authors:** Sujit Kumar, Vinayaka H. Damle, Tatyana Bendikov, Anat Itzhak, Michael Elbaum, Katya Rechav, Lothar Houben, Yaakov Tischler, David Cahen

**Affiliations:** † Dept. of Mol. Chem. & Mater. Science, Weizmann Inst. of Science, Rehovot 7610001, Israel; ‡ Bar-Ilan Inst. for Adv. Mater. & Nanotech. & Dept. of Chem., Bar-Ilan Univ., Ramat Gan 5290002, Israel; § Dept. of Chem. Research Support, Weizmann Institute of Science, Rehovot 7610001, Israel; ∥ Dept. of Chem. Biol. Physics, Weizmann Institute of Science, Rehovot 7610001, Israel

**Keywords:** 2D/3D, halide perovskite, vapor-phase growth, ultrathin, in situ monitoring

## Abstract

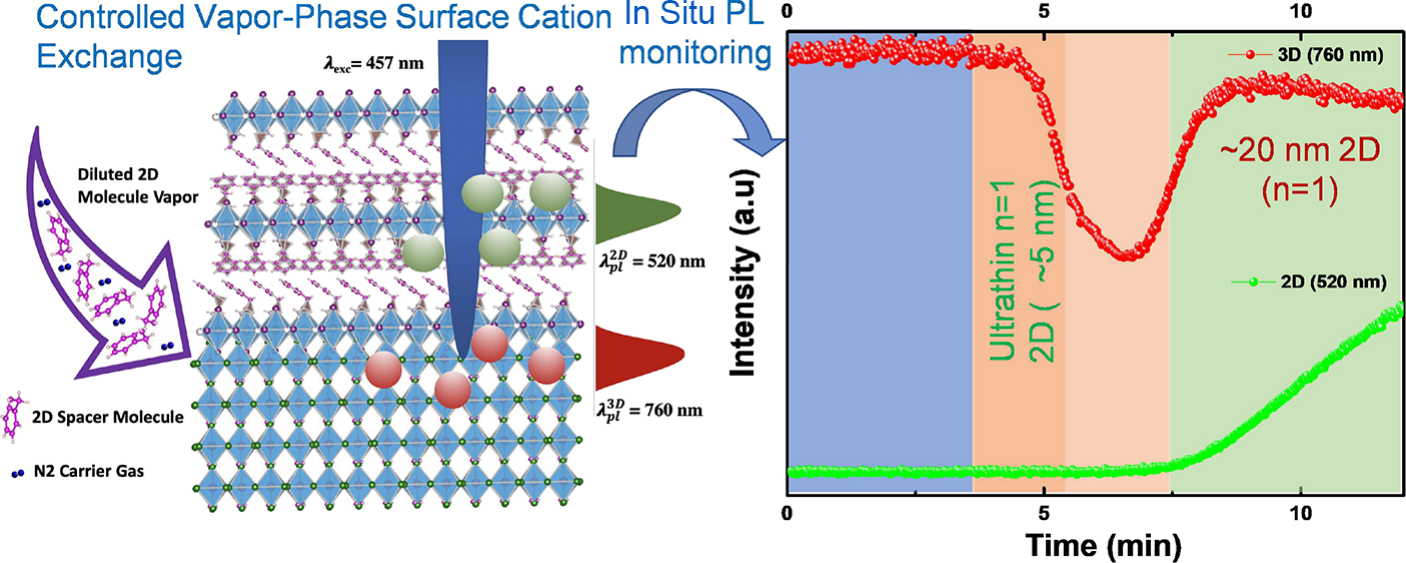

Two-dimensional (2D) halide perovskites, HaPs, can provide chemical stability to three-dimensional (3D) HaP surfaces, protecting them from exposure to ambient species and from reacting with contacting layers. Both actions occur with 2D HaPs, with the general stoichiometry R_2_PbI_4_ (R: long or bulky organic amine) covering the 3D ones. Adding such covering films can also boost power conversion efficiencies of photovoltaic cells by passivating surface/interface trap states. For maximum benefit, we need conformal ultrathin and phase-pure (*n* = 1) 2D layers to enable efficient tunneling of photogenerated charge carriers through the 2D film barrier. Conformal coverage of ultrathin (<10 nm) R_2_PbI_4_ layers on 3D perovskites is challenging with spin coating; even more so is its upscaling for larger-area devices. We report on vapor-phase cation exchange of the 3D surface with the R_2_PbI_4_ molecules and real-time *in situ* growth monitoring by photoluminescence (PL) to determine limits for forming ultrathin 2D layers. We characterize the 2D growth stages, following the changing PL intensity–time profiles, by combining structural, optical, morphological, and compositional characterizations. Moreover, from quantitative X-ray photoelectron spectroscopy (XPS) analysis on 2D/3D bilayer films, we estimate the smallest width of a 2D cover that we can grow to be <5 nm, roughly the limit for efficient tunneling through a (semi)conjugated organic barrier. We also find that, besides protecting the 3D against ambient humidity-induced degradation, the ultrathin 2D-on-3D film also aids self-repair following photodamage.

## 1 Introduction

Halide perovskites, HaPs, with ABX_3_ stoichiometry (A: MA^+^, FA^+^, Cs^+^; B: Pb^2+^, Sn^2+^, Ge^2+^; X: Cl^–^, Br^–^, I^–^) are semiconductors for optoelectronics that are vigorously studied, especially over the last decade.^1,2^ This effort led to their excellent performance as active layers in solar cells and light-emitting diodes, as well as in lasers and radiation detectors. Significantly, the power [solar (photon) → electrical] conversion efficiency (PCE) of HaP-based solar cells is approaching those of the best silicon-based PV devices with over 25.7% PCE values for small (<0.1 cm^2^) laboratory devices.^3^

Despite the exceptional optoelectronic attributes of the HaPs, well suited for different device applications, their long-term stability remains a severe source of concern *en route* to commercialization.^4^ Two-dimensional (2D) R_2_BX_4_ HaPs, which are structural analogues of their three-dimensional (3D) ABX_3_ counterparts, have gained much attention over the last few years as a viable route to impart improved functional and chemical stability to 3D layers by covering the latter, also in device configurations.^5,6^ The commonly used 2D perovskites are of the Ruddlesden–Popper (RP) R_2_A_*n*–1_B*_n_*X_3*n*+1_ type, where the bulkier organic cations (R in R_2_A_*n*–1_B*_n_*X_3*n*+1_) separate the metal-halide (inorganic) octahedral sheets and ″*n*″ is the number of such inorganic sheets stacked together. The price for the improved stability is wider 2D than 3D band gap and poorer charge separation within the material (higher exciton binding energy).^7,8^

Using 2D perovskites as capping layers for 3D absorbers in a PV configuration combines the superior 2D chemical stability with the excellent optoelectronic properties of the 3D perovskites.^9^ Thus, this combination has and is actively pursued and has led to both highly efficient and stable HaP-based solar cells. Most 2D-on-3D HaP layers showing high photovoltaic efficiencies use wet-chemical processing, i.e., spin-coating of 2D precursors, i.e., the organo-amine or its (halide) salt, dissolved in a suitable solvent, onto an existing 3D HaP film, followed by annealing the resulting composite film at ∼100 °C.^10−13^ The solvent (IPA) that is used to dissolve 2D organic ligands inevitably leads to reconstruction of the 3D surfaces,^14^ including PbI_2_-rich domains, long-term implications of which are still not very clear.^15^ Furthermore, this method is not optimal if an ultrathin 2D layer (<10 nm) has to be deposited uniformly and conformally to form a continuous moisture barrier on the 3D surfaces. Ultrathin 2D layers on 3D HaP surfaces are essential to minimize the efficiency losses in PV devices arising due to the poor out-of-plane charge transport across these layers that consist of hydrophobic alkyl or aryl tail of the amine, R. In addition, for 2D–3D HaP bilayers with unfavorable energy-level alignment for electron/hole transport, the use of an ultrathin 2D will minimally hinder the charge extraction to the respective (E/H)TL-metal electrodes.

Despite various approaches to control the 2D/3D interfaces, phase purity of the top 2D layer remains a challenge.^16−18^ The formation of mixed 2D phases characterized by the presence of multiple emission/absorbance peaks in the optical measurements can be attributed to fast reaction rates (a few seconds) involved in these 2D surface treatment processes, which enable kinetically controlled products (higher *n* phases)^19−21^ to form in addition to the thermodynamically stable *n* = 1 2D phase.^22^ Such suboptimal top layer quality can result in recombination losses in devices due to the inhomogeneous staggered spatial distribution of quasi-2D (mixed 2D–3D) phases across the 2D/3D interface.

Given the potential that 2D HaPs have for modifying the 3D surfaces and the wide chemical tunability of the side chain of the amine cation (that forms the 2D layers),^23^ we searched for alternative routes to achieve ultrathin, uniform, and phase-pure 2D layers on 3D HaP surfaces. Here, we show a controlled vapor-phase, topotactic growth approach for depositing 2D, covering layers on polycrystalline 3D layers by relying on the chemistry at the 3D HaP surfaces, where the width of the 2D cap can be controlled to ultrathin regimes (∼5 nm). The characteristic photoluminescence (PL) emission of the 2D and 3D HaPs enables *in situ*, real-time monitoring of the R-cation-exchange process at the 3D surfaces during their controlled exposure to the organo-amine molecules. This was achieved in a custom-built growth chamber where the slow vapor-phase process of 3D to 2D surface conversion appears to aid the formation of the thermodynamically stable *n* = 1 2D phase on the 3D HaP layers. The time evolution of the PL emission from the HaP shows the progressive transition of the 3D MAPbI_3_ film surface into 2D FPEA_2_PbI_4_/3D MAPbI_3_ heterostructure, where FPEA^+^ is the fluorinated phenethylammonium 2D HaP-forming cation. The PL intensity (of 2D and 3D HaP emission bands) vs growth time then guides when to terminate the process to achieve 2D capping on the 3D layers. The 2D/3D heterostructure layers that resulted in the different 2D growth regimes were characterized using X-ray and electron diffraction (for structural) and photoelectron spectroscopy (for chemical composition and electronic properties). In addition, the films’ optical and morphological characteristics were assessed for ultrathin 2D-on-3D layers. We find that even a <5 nm 2D FPEA_2_PbI_4_ layer can sufficiently stabilize the underlying MAPbI_3_ perovskite against moisture-induced degradation. As an additional advantage, we show from the PL damage and recovery measurements that the top 2D layer helps faster recovery of mild to moderate photodamages in 3D perovskite domains, exhibiting a hitherto unexplored benefit of having an ultrathin 2D capping on 3D MAPbI_3_ surfaces.

## 2 Results and Discussion

### 2.1 *In Situ* PL Monitoring to Track 2D Growth on 3D HaP Surfaces

The polycrystalline films of 3D MAPbI_3_ perovskites were deposited on either glass or fluorine-doped tin oxide (FTO) substrates using an optimized recipe described in the Experimental Section in the Supporting Information (SI). The 3D HaP films were exposed to vapors of F-PEA molecules in N_2_ as a carrier gas to initiate the surface cation exchange reactions wherein the longer FPEA^+^ ions replaced the MA^+^ ions within the 3D HaP lattices. The rate of these reactions was controlled with two flowmeters, allowing independent manipulation of the flow rates of the gaseous precursors and the N_2_ carrier gas into the 2D growth chamber. The custom-built chamber was designed with perforated walls that connect the chamber to the inlet and outlet gas feedlines (Figure S1b). This assured uniform exposure of the 3D HaP surfaces to the reactant vapors. A transparent viewport was added to the chamber, which enabled investigating the change in photoluminescence from the film’s (near) surface regions during the reactions. In this study, the pure MA cation-based 3D HaP was used as the growth substrate instead of (MA, FA, Cs)-, (Br, I) perovskites,^24,25^ which can yield more efficient solar cells because especially the presence of multiple cations that can exchange on 3D surfaces complicates interpreting the results in terms of homogeneity of surface conversion reactions and affects the ability to yield phase-pure *n* = 1 2D growth and is likely to require further study to yield phase-pure *n* = 1 2D growth.

**Figure 1:**
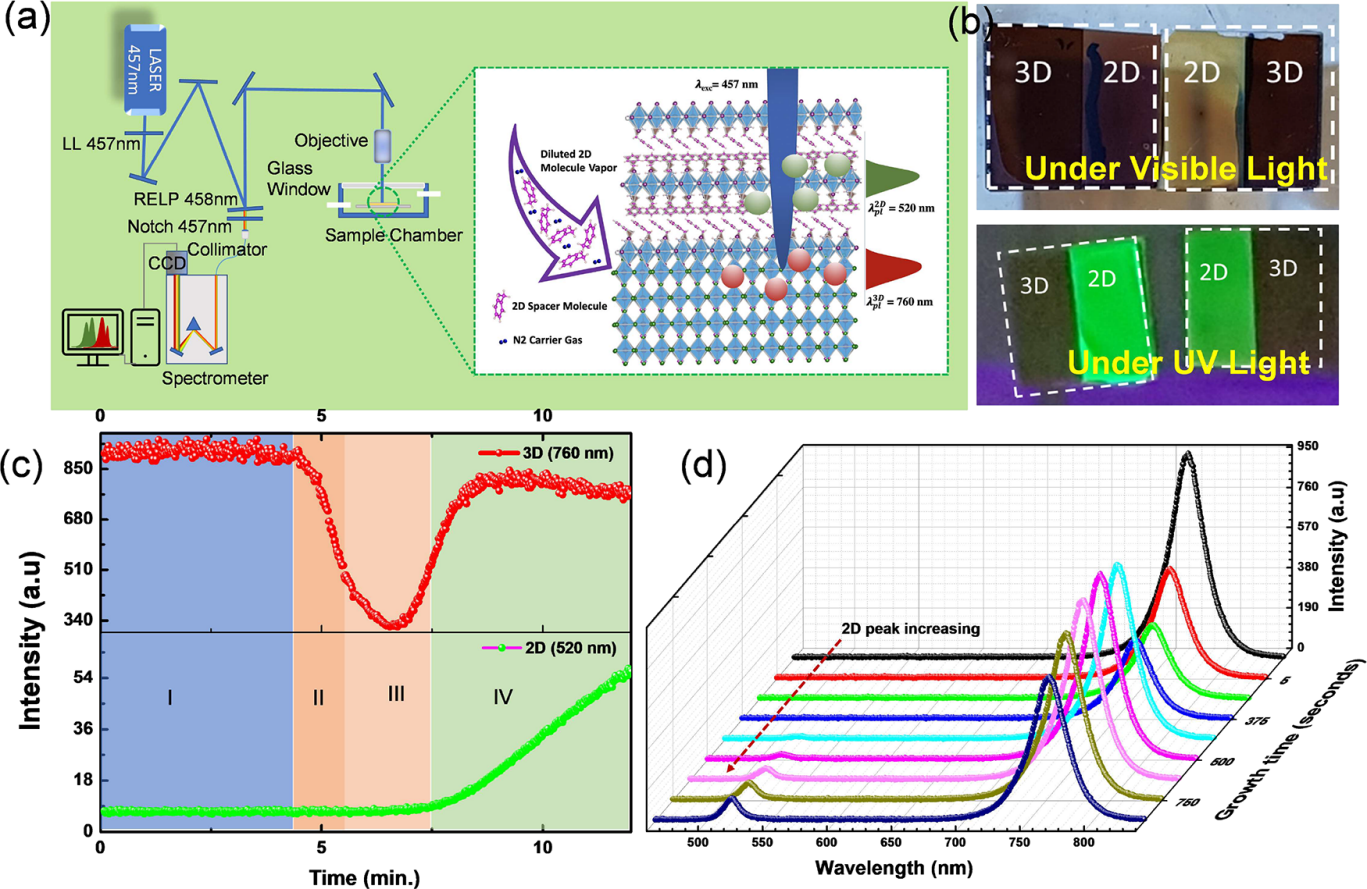
*In situ* PL measurements. (a) Schematic showing vapor-phase surface conversion of 3D to 2D HaP with the excitation and collection optics for operando PL measurements. For the actual setup, see Figure S1 in the SI. (b) Example of 2D HaP growth on unmasked regions of 3D MAPbI_3_ perovskite films using vapor-phase surface conversion approach. The green emission from the 2D HaP can be seen clearly with UV illumination. (c) PL intensity vs time profile for 3D and 2D emission bands during the 2D growth on 3D surfaces. (d) Steady-state PL evolution at different times during the growth regimes as marked in (c), starting with regime I (first three traces from the back), regime II (4th, dark blue trace), regime III (5th and 6th, light blue and purple traces), and regime IV (most forward three traces).

Figure 1a shows a schematic of the vapor-phase 3D to 2D conversion with PL measurement optics, built to study the cation exchange reactions *in situ*. The actual home-built growth chamber, the gas flow lines, and the optical setup are shown in Figure S1. A primary criterion for growing ultrathin 2D layers onto 3D perovskite with a high optoelectronic quality of the 2D/3D interface (i.e., have phase-pure 2D layers) is to control and slow down the phase conversion reactions. This was readily possible in our approach, unlike what is the case with spin-coating, where conversion kinetics are much faster. Figure 1b, for example, shows two MAPbI_3_ samples, unmasked regions of which were exposed for 2D growth using the system shown in Figure 1a. Under UV light, bright green luminescence can be seen from these 2D regions. In Figure 1c, we show the PL intensity variation of 3D (∼at 760 nm) and 2D (∼520 nm) emission peaks as a function of reaction, i.e., 2D growth, time. The corresponding steady-state PL spectra are shown in Figure 1d. Different regimes of growth, which can be identified with the 3D → 2D transition, are seen from changes in emission spectra (Figure 1d). These different growth regimes are marked I–IV in Figure 1c.

In region I, the 3D PL emission intensity remains stable primarily as there is no in-flow yet of FPEA molecules into the growth chamber, and only N_2_ flows over the 3D HaP sample. Region II marks the onset of the surface chemical reactions between MAPbI_3_ and the FPEA molecules after the precursor vapor line is opened and set to an optimum flow rate. In this region, we observe a decrease in MAPbI_3_ PL intensity, but there is still no detectable 2D PL emission signal. Presumably, even if there is already a 2D growth in this region, it is too thin to yield an emission signal that is detectable with our measurement system. We surmise that the decrease in the 3D PL emission results from collapsing of [PbI_6_]^4–^ octahedrons in the outermost layers of the 3D HaP lattice due to the loss of MA^+^ ions as methylamine (gas). The deprotonation of the MA^+^ cations to MA in the presence of a higher concentration of FPEA molecules (than of MA) at the reaction (MAPbI_3_) surface follows the law of mass action where MA and FPEA both are highly basic and have strong tendencies to accept a proton. The proton that remains (to maintain electrostatic balance) then protonates FPEA to FPEA^+^, according to
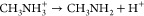
1
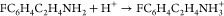
2where CH_3_NH_3_^+^ and FC_6_H_4_C_2_H_4_NH_3_^+^ are the molecular formulas for MA^+^ and long FPEA^+^ cations, respectively.

Therefore, the overall transformation reaction for such 3D → 2D conversion in the presence of FPEA molecules can be denoted by the following equation
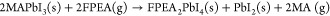
3

According to eq 3, two 3D MAPbI_3_ units are converted into one unit of 2D FPEA_2_PbI_4_ perovskite, with PbI_2_ as the co-product. Hence, the effective volume of the 3D MAPbI_3_ from which most of the PL emission signals are collected is reduced, i.e., the 3D PL intensity drops. The 3D to 2D conversion process is also expected to generate defects in the near-surface 3D domains where the reaction occurs due to bond breaking needed for the conversion. As a result, the system will go through a rapidly changing, dynamic state in which the nonradiative recombination will initially increase within what was the pristine 3D HaP. Thus, the observed drop in 3D PL intensity in regime II will also have a contribution due to the defect states formed by the conversion, within the 3D HaP, along with the part caused by the 3D mass loss that occurs due to the 3D to 2D conversion process. The decrease of 3D PL intensity continues throughout regime II, and, with decreasing slope into regime III, where the decrease saturates and starts to recover. Additionally, in this region, we observe the onset of 2D PL emission at ∼520 nm, which then increases during regime IV, where the 3D PL intensity saturates and starts to decrease mildly. These time-dependent changes in PL intensity indicate that optically detectable 2D layers now form on top of the 3D MAPbI_3_ films and that the 2D layer growth increases throughout regime IV, marked by increasing 2D and a slight decrease in 3D PL intensity. We can understand these results if the FPEA molecules diffuse into the 3D lattices and lead to their local collapse; the collapsed parts then reconstruct into the 2D HaP structure with long FPEA^+^ cations separating [Pb–I]_6_ octahedra. Hence, the 2D thickness grows into the 3D matrix (surface → bulk), resulting in the rise of the ∼520 nm 2D HaP emission intensity.

The increase in 3D PL intensity in regime (IV) is somewhat surprising because the 2D HaP can only form at the expense of the 3D layers, i.e., no extra volume is added. Furthermore, mass loss (of MA) implies that the 3D intensity (equivalent to the 3D fraction within the focal volume) should decrease. How, then, can the 3D PL recover in regime III?

The most probable explanation is that type-I heterojunction forms between the 2D and the 3D components, which allows the transfer of e–h pairs generated in the 2D film to the 3D part. There, the pairs are “stuck,” and their radiative recombination will yield the characteristic 3D PL emission. In addition, it is possible that there is a transfer of the energy absorbed by the 2D layer to the underlying 3D MAPbI_3_. A small part can also be due to 2D emission into the 3D region, where it can be absorbed and re-emitted.

Evidence for the explanations mentioned above is that the 3D PL emission enhancement (as shown in Figure S2a, SI) occurs specifically in spectral excitation regions where the 2D FPEA_2_PbI_4_ perovskite absorbs, i.e., 490–510 nm. Also, the early time evolution of 2D and 3D PL emission intensities after photoexcitation of the 2D/3D bilayer film, formed in regime IV (Figure S2b, SI), shows that the increase in 3D PL emission is accompanied by a decrease in the 2D emission. This anticorrelation is consistent with the energetic and/or electronic coupling between the two layers. This 2D → 3D radiative energy transfer was shown earlier by Song et al. in PEA_2_PbI_4_/MAPbBr_3_ HaP heterostructures using two photon-excitation PL microscopy.^26^ In our experiments, we find that this 2D to 3D energy/carrier transfer not only compensates for the loss of 3D PL emission due to the loss of its volume but also dominates the 3D emission as seen from the increase in its intensity in region III and partly through region IV. Further, as the reaction progresses in region IV, the 3D PL intensity drops (Figures 1c and S3), while 2D emission continuously increases. We suggest that such behavior occurs because there is an insufficient volume of 3D MAPbI_3_ within the optical focal volume to absorb the energy/carriers that are absorbed/generated in the 2D layers. The steady-state PL spectra (Figure 1d) of the 2D/3D HaP layers, measured in the different growth regimes, fit the trend in the intensity variation described above.

Another significant result is that with this slow surface cation exchange to prepare a 2D on 3D HaP structure, phase-pure 2D FPEA_2_PbI_4_, i.e., the *n* = 1 phase, is obtained with characteristic emission at ∼520 nm. In contrast, for wet chemically processed 2D layers on 3D HaPs, other quasi-2D (also called 2D–3D) phases with *n* = 2, 3, or even higher^16,17^ can form as the reaction product, in addition to the true *n* = 1 2D phase. Graded distribution of quasi-2D phases on/near 3D surfaces has been reported,^27,28^ but no control over their (cascade-like) arrangements was possible. Such compositionally graded systems are expected to have increased recombination losses at the interfaces. Our approach of growing 2D on 3D thus provides a feasible route to tune *n* = 1 phase-pure 2D overlayer widths (see also later for XPS results) on the 3D HaP to optimize them for use in devices, esp. solar cells.

### 2.2 Structural and Optical Properties of 2D-on-3D HaPs in Different Growth Regimes

**Figure 2:**
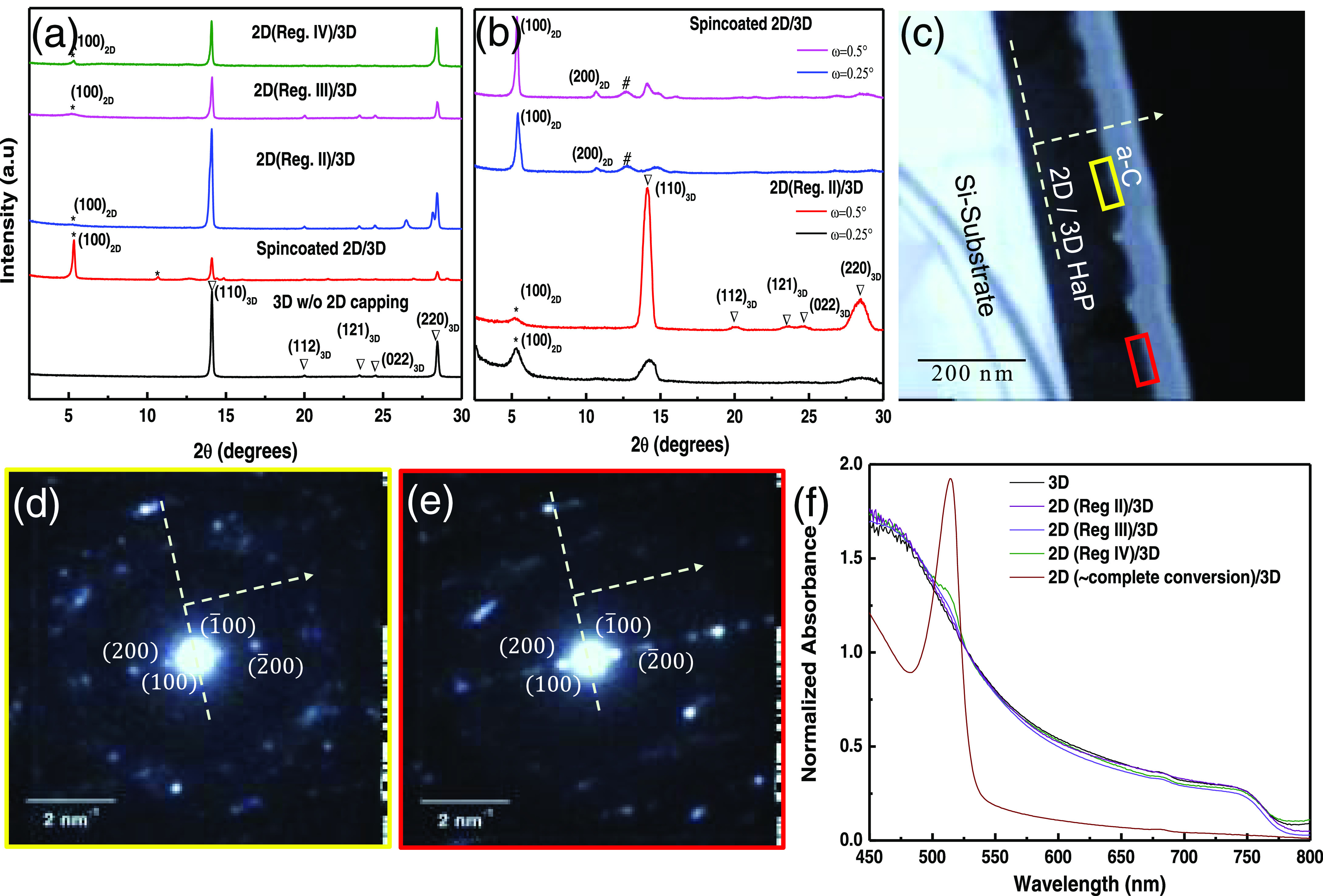
Structural and optical absorbance characteristics of 2D/3D bilayers. (a) Bragg–Brentano (θ–2θ) and (b) grazing incidence XRD scans for 3D, spin-coated, and vapor-phase grown 2D-on-3D MAPbI_3_ perovskite layers. (c–e) Four-dimensional scanning transmission electron microscopy (4D STEM) scanning nano-diffraction data: (c) virtual bright-field image of a 2D/3D bilayer with 2D layer, grown in regime IV of the “PL intensity vs time profile,” shown in Figure 1c; (d, e) locally averaged electron diffraction patterns from the specific surface areas marked in yellow and red in (c). The characteristic diffraction spots for 2D HaP lattice planes are suitably marked. The basal plane (100) reflections of the 2D *n* = 1 phase occur at ∼0.6 nm^–1^ in reciprocal space, indicative of the larger, ∼1.6 nm interplanar (100) spacing in the 2D FPEA_2_PbI_4_ phase than the 0.63 nm interplanar spacing of the 3D (110) planes. The diffraction patterns in (d, e) have been rotated to match the scan coordinate system in real space such that the white dashed lines in (d) and (e) indicate the substrate plane and the corresponding substrate normal shown in (c). (f) UV–vis optical absorbance of 2D/3D bilayer films, prepared by controlled vapor-phase surface reaction of 3D MAPbI_3_ layers.

Figure 2 shows the X-ray diffraction patterns of the MAPbI_3_ thin films and those treated with FPEA vapors for different durations in the 2D growth chamber, i.e., in regimes II, III, or IV (regime I represent untreated MAPbI_3_ films). The pristine MAPbI_3_ films exhibit peaks corresponding to diffraction from the tetragonal (110), (112), and (220) planes at 2θ values of roughly 14, 20, and 28° (for Cu Kα X-radiation). When exposing the 3D HaP surface to the FPEA vapors, the 2D phase with its characteristic larger interplanar (*d*-spacing) forms over the 3D surfaces. The signatures of this growth are seen in the *in situ* PL measurements, shown in Figure 1. However, for 2D/3D bilayers with 2D growth terminated in regime II, the θ–2θ XRD scans do not show the presence of 2D domains. XRD evidence for 2D formation starts to be seen in regime III, and more so in regime IV, as new diffraction peaks appear at smaller 2θ values (larger interplanar spacing) of ∼5.4°.

The apparent inconsistency between the PL and XRD results on samples in regime II possibly relates to 2D domains that are too small to yield any measurable diffraction in the Bragg–Brentano XRD geometry. To check this, we performed grazing incidence (incident angles of 0.25 and 0.5°) XRD measurements to enhance the surface signal. The results show the small 2θ (∼5.4°) reflection of the larger lattice spacing of the 2D HaP, indicative of the 3D → 2D surface transformation unequivocally.

To compare 2D layers formed via conventional spin-coating of FPEAI solution with those formed by vapor-phase surface cation exchange, we also show θ–2θ and grazing incidence XRD scans of the spin-coated films in Figure 2a,b, respectively. The XRD scans for the spin-coated 2D-on-3D layers show strong 2D reflections (while 3D reflections are weaker at 14.1°) at ∼5.4 and 10.8° 2θ values, indicating relatively thick 2D domains on the 3D MAPbI_3_ layers. Additionally, the XRD measurements show evidence for PbI_2_ with its characteristic reflection at 2θ = 12.7°. Furthermore, the 2D layers are noticeably thicker with spin-coating than with the vapor-phase exchange method. Thicker 2D films reduce the charge transport efficiency across 2D/3D interfaces, a critical issue for current passing devices that appears solved with the controlled vapor-phase exchange. We do not observe PbI_2_ XRD signatures in vapor-phase grown 2D layers on 3D MAPbI_3_ surfaces (Figure 2a,b), while PbI_2_ appears as a co-product in the MAPbI_3_ surface conversion by reaction 3. A possible reason can be that the PbI_2_ domains and other 2D species that might form by possible PbI_2_-FPEA intercalates, as PbI_2_ has a layered (hexagonal, *P*63*mc*) structure, are incoherent and do not diffract the X-rays sufficiently to produce measurable intensities by the detector, especially when the grown 2D FPEA_2_PbI_4_ layer is ultrathin.

Nanoscale information of the 2D/3D bilayers, grown with the vapor-phase cation exchange method, was obtained using a scanning nanobeam electron diffraction experiments^29^ (a variant of 4D STEM, see the Experimental Section in the SI for details), performed at a very low electron fluence (1–2 e/(Å^2^ ms)^−1^) to minimize damage on these beam-sensitive samples. Figure 2c shows a virtual bright-field image of a 2D/3D heterostructure with the 2D layer grown in regime IV of the PL intensity vs growth profile in Figure 1c. The image was obtained by mapping the nondeflected e-beam intensity for each point of scanning (few nm^2^) on the sample that shows distinct contrast features for the Si substrate, the 2D–3D HaP region and the carbon protective coat. However, the 2D coat is not distinguishable from the 3D HaP in the bright-field image. The electron diffraction patterns (EDPs) collected for each scan position on the sample store the structural information of crystalline lattices that diffract the e-beam. Two such EDPs from different near-surface regions (near to the interface with carbon coat) of the 2D/3D lamella are shown in Figure 2d,e. The areas from the sample in real space from which the diffraction patterns have been collected are marked in Figure 2c by red and yellow boxes. The primary diffraction spots, characteristic of the (00*l*) crystallographic planes of the 2D HaP lattices, are marked in the diffraction patterns (Figure 2d,e). The diffraction patterns are suitably rotated to compensate for the rotation relative to the scan coordinate system in real space so that dashed white arrows can identify the normal to the substrate (Si) plane in Figure 2d,e. Notably, both diffraction patterns show the presence of a 0.6 nm^–1^ diffraction spot in the reciprocal space, which translates into an interplanar distance of ∼1.6 nm in real space. This unambiguously shows that the surface of the 3D HaP has been reconstructed by the controlled exposure to vapors of long organic amines (FPEA in this case) into a 2D HaP structure with its characteristic higher interplanar spacing between the [PbI_6_]^4–^ octahedra than in the 3D MAPbI_3_ structure. Furthermore, the EDPs do not exhibit the presence of higher “*n*” phases (*n* > 1), and, hence, together with the results of PL and XRD measurements (Figures 1d and 2a,b respectively), it appears that phase-pure *n* = 1 2D formed on top of the 3D perovskite using our 2D growth method. Additionally, Figure 2d,e shows that the (00*l*) diffraction spots lie nearly parallel to the substrate normal, marked by white dashed arrows, meaning that the (00*l*) 2D planes are oriented horizontally along the Si/3D HaP substrate plane. This is consistent with the XRD results (Figure 2a,b) and shows that the 2D (00*l*) planes have a preferred orientation along the primary (110) crystallographic planes of the 3D HaP lattices. This observation points to a topotactic transformation from the primary (110) planes of the 3D HaP lattice to the 2D structure.

Further, to observe the effect of the as-grown 2D layers with different thicknesses, UV–vis absorption spectra of 2D/3D bilayer films were measured, the results for which are shown in Figure 2f. All samples show similar absorption spectra with onsets at ∼770 nm, representing the absorption edge of MAPbI_3_. However, slight differences in the spectral features can be discerned in the 500–530 nm wavelength range. For comparison, the UV–vis absorption of a thick 2D layer, i.e., with nearly complete conversion of the MAPbI_3_ by FPEA molecules, is also shown. Based on the latter spectrum, the changes in the 500–530 nm spectral range of 2D/3D bilayers are consistent with the absorption peak of the *n* = 1 FPEA_2_PbI_4_ 2D layer. This suggests that thin 2D HaP layers are indeed forming a capping on 3D MAPbI_3_ surfaces, consistent with the XRD measurements. While the converted film is too thin in region II to induce any change in the MAPbI_3_ absorption spectrum, the 2D absorption becomes clearly observable in regions III and IV of the PL intensity vs time plot (Figure 1c).

### 2.3 X-ray Photoelectron Spectroscopy Measurements and Estimation of the Lower Bound for 2D Layer Thickness

XPS measurements were performed on pure 3D HaP and 2D/3D bilayer film prepared with different 2D growth durations. The fluorine atom, attached to the phenyl ring of the FPEA, helps to differentiate qualitatively between the two chemically correlated perovskite phases, FPEA_2_PbI_4_ and MAPbI_3_.

**Figure 3:**
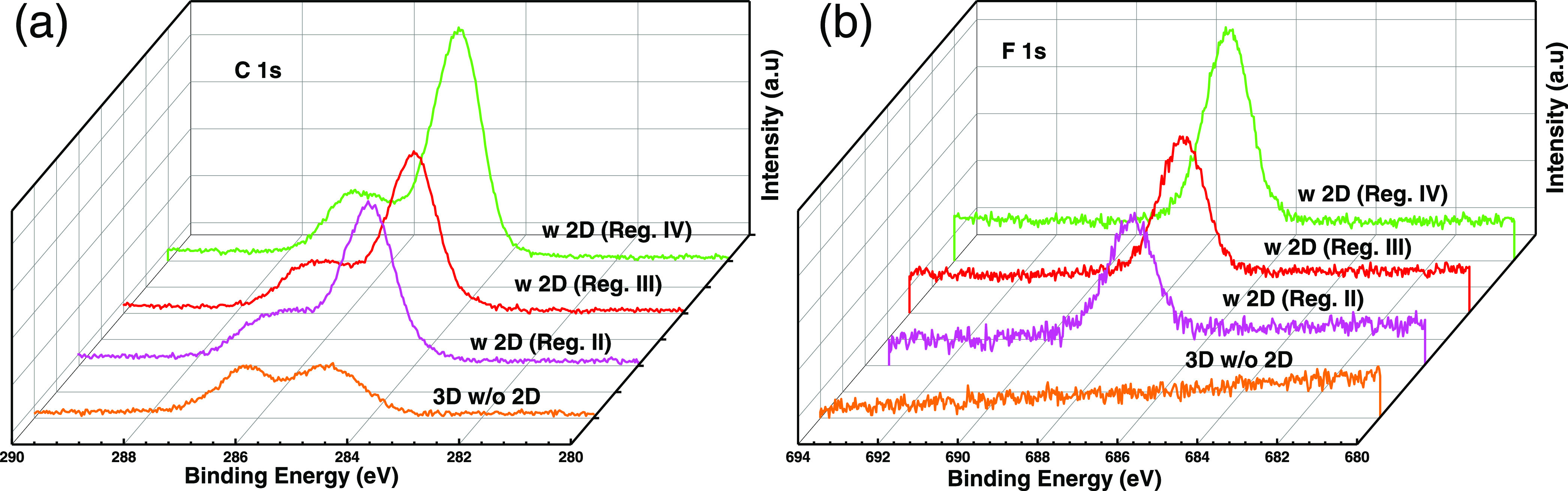
High-resolution XPS spectra of the C 1s (a) and F 1s (b) regions of 3D and 2D-on-3D perovskite films for 2D growth, with growth terminated in different regions of PL intensity vs time profile as shown in Figure 1c. The F 1s signal appears only for layers that have (sufficiently thick) 2D FPEA_2_PbI_4_ overlayers on the MAPbI_3_ perovskite film, as is seen in (b).

The XPS binding energy (BE) peaks of the photoelectrons emitted from the C 1s, N 1s, I 2p, Pb 4f, and F 1s core levels are shown in Figures 3 and S4 (SI). All of these can, and, in the case of the ultrathin vapor-phase deposited 2D films, likely will have contributions from both 2D and 3D components in the 2D/3D bilayer films. The F 1s BE peak (Figure 3b) at ∼686 eV is attributed to photoelectrons from the fluoro-phenyl ring in the FPEA^+^ cation of the 2D capping layer, which is obviously absent for the pure MAPbI_3_ layers without a 2D capping.

Quantitative estimation of the 2D width in the 2D/3D bilayers is, however, challenging. For example, the cross-sectional scanning electron microscopy images of the 2D/3D bilayers, shown in Figure S5 (in SI) for 2D layers grown in regimes II and III (of Figure 1c), do not clearly distinguish the thin 2D overlayers from the 3D MAPbI_3_ substrates on which they grow. Only the entire bilayer stack thickness can be measured to be ∼300 nm. However, for relatively thicker 2D layers, e.g., those grown in regime IV of the PL intensity vs time profile (Figure 1c), the cross-sectional scanning electron microscopy (SEM) image (Figure S5c) shows hazy signatures of ∼20 nm 2D capping on top of the 3D perovskite layer. To add, the van der Waals nature of the forces between the large organic cations and the sheets of inorganic Pb–I octahedra makes them highly susceptible to structural damage from high energy (up to hundreds of keV) probes, such as electron beams in transmission electron microscopy. To prevent/minimize sample degradation and reliably measure the 2D layer widths in their functionally active states, we analyzed the intensities of core-level photoelectrons (and the atomic concentration ratios) of 2D and the 3D components in 2D/3D HaP bilayers and compared them with the relevant atomic percentages, based on their chemical structure. The detailed calculations for the 2D layers, grown in regimes II and IV, appear in Section S2 in the SI. For regimes III and IV, the 2D layer thickness appears to be ≥10 nm, i.e., too thick (well beyond the (Al Kα) X-ray penetration depth) to determine the 2D overlayer widths reliably. For the 2D capping layers grown in regime II, our calculations suggest ca. 4–5 nm widths. This finding is at the high end of a barrier (at least for conjugated organics) that allows efficient tunneling for ∼1 sun photogenerated carriers^30^ (in a solar device with 2D capping layer) across it, in this case, the 2D moisture barrier (which is electrically more resistive than the 3D substrate).

### 2.4 2D/3D Heterointerface Energetics and Its Effect on Photocarrier Transport

The energy band alignment at the 2D/3D interface is important for the overall current transport characteristics of the system. In a photovoltaic device, nearly all carrier photogeneration will be in the 3D HaP, but one of the contacts will be with the 2D overlayer. Thus, energy-level (mis)alignment can (break)make a device. To get information on this aspect, we performed ultraviolet photoelectron spectroscopy (UPS) on the 3D films and 2D/3D ones with a relatively thick 2D layer, i.e., grown in regime IV of Figure 1c, deposited on ITO/glass substrates.

**Figure 4:**
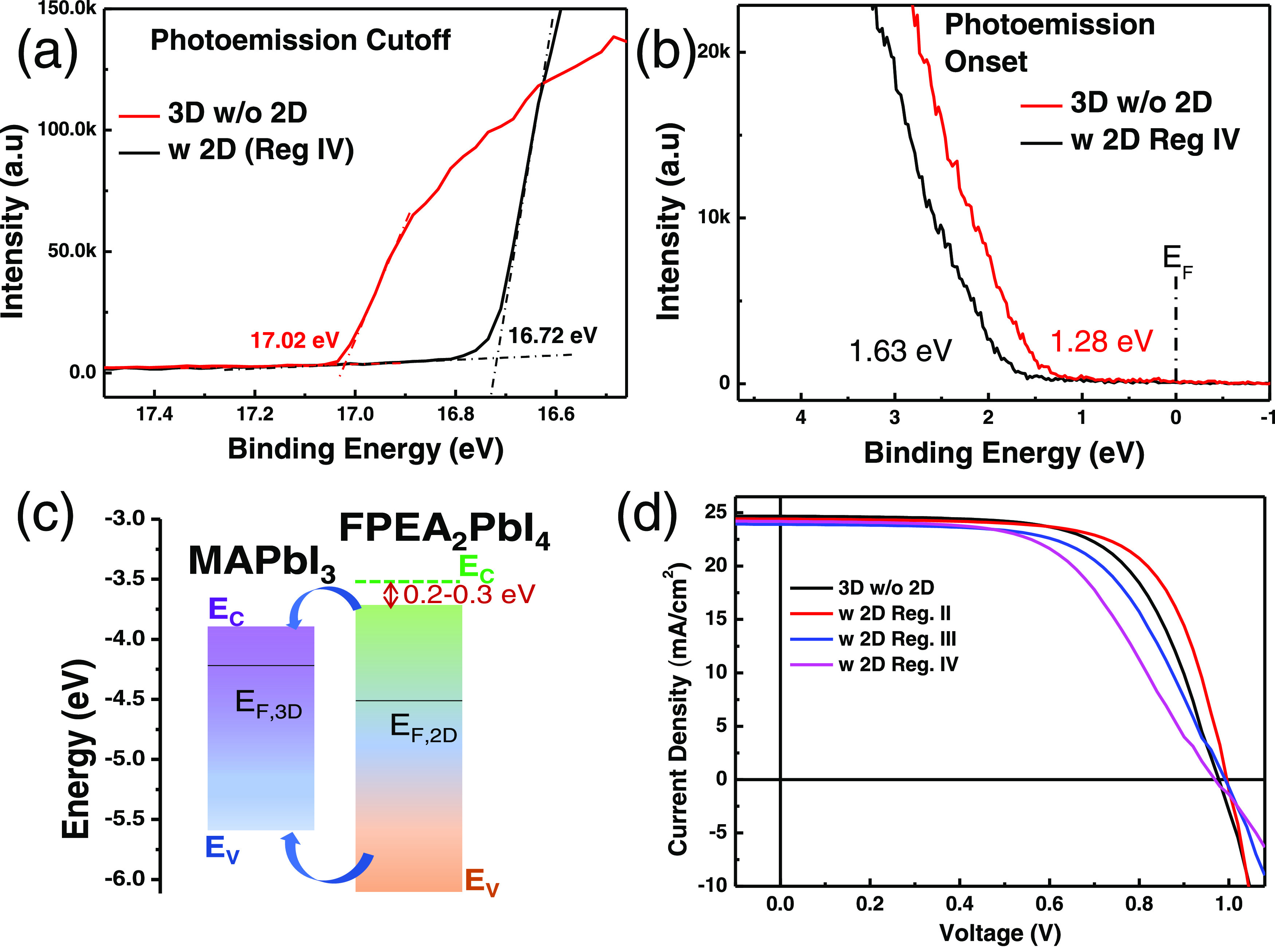
UPS measurements and the derived energy-level positions of the 2D and 3D HaP layers. (a) Secondary photoelectron cutoff energy (*E*_cutoff_) region from which the work function (*W*_F_) values are derived. (b) UPS data near the Fermi energy (*E*_F_) with the derived VBM values. (c) Energy-level alignment around the Fermi level, *E*_F_ (marked by bold black line within 3D and 2D band gaps), with all energies relative to the local vacuum level; *E*_C_ and *E*_V_ are the conduction band minimum and valence band maximum, respectively. In this scheme, the PL-derived band gap is used, i.e., without correction for the exciton binding energy in the 2D HaP, which has been estimated at 200–300 meV.^31,32^ The green dashed line above the FPEA_2_PbI_4_ energy levels indicates the *E*_C_ position after considering the 2D exciton binding energy. For detailed UPS analysis, see Section S3 in the SI. (d) *J*–*V* characteristics of photovoltaic devices fabricated with 3D and vapor-phase grown 2D-on-3D HaP films in the device configuration: ITO/SnO_2_/3D MAPbI_3_/2D FPEA_2_PbI_4_/spiro-OMeTAD/Ag.

The results shown in Figure 4a,b are relevant for determining the work functions (*W*_F_) to get the Fermi level (*E*_F_) energies relative to the vacuum level and the valence band maximum (VBM) energies with respect to *E*_F_. The full UPS spectra of the 3D and 2D/3D bilayers are shown in Figure S6, and the details pf the *W*_F_ and VBM values calculations are given in Section S3 in the SI. The *W*_F_ values relative to the vacuum levels were estimated to be 4.2 and 4.5 eV for pure 3D and 2D-on-3D bilayer films, respectively. The valence band maxima for the corresponding films were determined from the onset of the leading edges of the spectra shown in Figure 4b. The ionization energies (IE) for the 3D and 2D/3D films were calculated (using the *W*_F_ and VBM values) to be 5.5 and 6.1 eV, respectively. The conduction band minima (CBM, *E*_c_) were then approximated using the optical band gap values obtained from the PL spectra of 3D and 2D HaP films. The energy band alignments for MAPbI_3_ in contact with a 2D FPEA_2_PbI_4_ layer are shown in Figure 4c. However, due to the order of magnitude higher exciton binding energy in the 2D than in the 3D perovskite (hundreds vs tens of meV), its electronic band gap should be at least a few hundred meVs wider than the optical band gap, deduced here from the PL measurements; the increased band gap means that the *E*_C_ moves by 0.2–0.3 eV toward the vacuum level energy.^8,31,32^ With this correction (see the green dashed line on FPEA_2_PbI_4_ energy levels in Figure 4c), we arrive at a picture that makes it even more apparent that the relative *E*_C_ and *E*_V_ energy levels lead to a type I junction between the two layers. Such a junction has a large uphill energy barrier for extracting photogenerated holes from MAPbI_3_ toward the hole-selective contacts (typically, Spiro-OMeTAD in laboratory PV cells). This will, if the tunneling through the 2D is inefficient, cause recombination losses at the HaP/hole-selective contact interfaces in a photovoltaic device. Though the barrier height for electron transport across the 3D to 2D interfaces is smaller than for holes, electrons moving toward the hole transporting layers will still be blocked, which is important for functional devices, such as solar cells.

To assess the photogenerated carrier dynamics in the 2D/3D perovskite heterostructure, with what we estimate to be >0.5 eV barrier for hole extraction from the 3D via the 2D capping layer, we studied the time-resolved PL decay of 760 nm emission of the 3D HaP with different thicknesses of the 2D layer on top. The corresponding PL decay curves and the lifetimes, extracted by fitting the data to a mono-exponential decay, are shown in Figure S7 in the SI. For ultrathin (≤∼5 nm) 2D-on-3D bilayers, the 3D decay lifetime improves slightly from ∼13 to 17 ns, which can be ascribed to the previously reported 2D passivation of nonradiative recombination centers at the 3D surfaces. However, for thicker 2D layers, i.e., those grown in regimes III and IV, we see that the 3D decay lifetimes decrease substantially, reducing to 5 ns for >10 nm 2D layers (in regime IV). While this reduction of carrier lifetime in 3D layer in the presence of the 2D overlayers may at first be surprising, it is readily understood from the energy-level alignment between the 3D and 2D HaPs valence (and conduction) band edges, as shown in Figure 4c. In addition to the significant energy barrier for hole extraction from 3D to 2D layers, the conduction band minima energies block electron transfer (from 3D to 2D). The reason is that the 2D HaP electronic band gap will be at least a few hundred meVs higher than the optical band gap^31,32^ that was used to calculate the energy-level positions shown in Figure 4c. Thus, the photogenerated carriers will be confined within the 3D region. In addition, the excess photogenerated carriers within relatively thicker 2D layers (>10 nm) can now be transferred into the 3D regions due to the favorable energy-level positioning of the conduction and valence band edges of the 2D HaP layers relative to those of the 3D one. Such a process further populates the 3D bands with excess carriers, as a result of which the radiative recombination rate will increase. Hence, the carrier lifetime for the 3D layer will decrease for bilayer structures as the width of the 2D-on-3D cap grows.

Our results are consistent with that for 2D capping layer widths <5 nm, 3D carrier lifetimes remain practically unaffected by the unfavorable 2D vs 3D energy-level positioning (apart from the effect of 2D passivation of 3D surface defects). The energy barrier at the 2D/3D interface for hole extraction is circumvented by reducing the 2D thickness to where quantum mechanical tunneling of the normal ∼AM1.5 photocurrents is sufficiently efficient. The observed reduction in the carrier lifetime for thicker (than 5 nm) 2D layers on the 3D HaP films is consistent with the increase in the PL intensity from the 3D HaP. We ascribe this result to the extra carriers/energy, now being pumped into the 3D domains, where they recombine radiatively, thereby increasing the 3D PL emission intensity.

Further, the photovoltaic performance of the vapor-phase grown 2D-on-3D HaP layers was briefly checked by fabricating PV devices in n–i–p configurations: indium tin oxide (ITO)/tin oxide (SnO_2_)/3D MAPbI_3_/2D FPEA_2_PbI_4_/spiro-OMeTAD/Ag (see the Experimental Section for details). The current density–voltage (*J*–*V*) characteristics shown in Figure 4d were obtained under AM 1.5G solar illumination. The detailed variation of device efficiencies, short-circuit current density (*J*_SC_), open-circuit voltages (*V*_OC_), and fill factor (FF) for over 15 PV devices are shown in Figure S8 in the SI. The PV analysis for these devices shows that for the ultrathin 2D layer (i.e., those grown in reg. II), the *n* = 1 2D overlayer benefits the device power conversion efficiency (PCE) primarily due to the improvement in *V*_OC_ and FF. The maximum PCE obtained for such 2D/3D layers is ∼16.7%, 1.07× the 15.6% for the control devices without 2D overlayers. The increase in *V*_OC_ and FF for the 2D/3D bilayers implies an effective passivation of nonradiative recombination of charges due to 3D surface defects and aligns well with the improved carrier lifetimes obtained for the HaP films alone, from the time-resolved PL studies. With 2D layers, which are ≥10 nm thick (reg. III, IV), the device efficiencies are significantly affected, primarily due to the decrease in FF values. Such behavior observed in this study could be understood from the poor charge transporting efficiency under bias, across too thick a pure *n* = 1 2D phase. To that reason can be added a significant barrier for photogenerated hole extraction from the 3D MAPbI_3_ layers, as deduced from the UPS measurements (Figure 4). It will be worth investigating the effect of 2D capping layer width and its phase purity on the PV performance of 2D/3D HaP-based PV cells and elucidating the principal causes of any such effect. We note, in passing, that an *n* = 1 2D phase, while ideal to impart maximum chemical stability to the 3D HaP underneath, is worse than any phases with *n* > 1 in terms of out-of-plane charge transport.

### 2.5 Surface Homogeneity of Ultrathin 2D-on-3D HaP Layers

**Figure 5:**
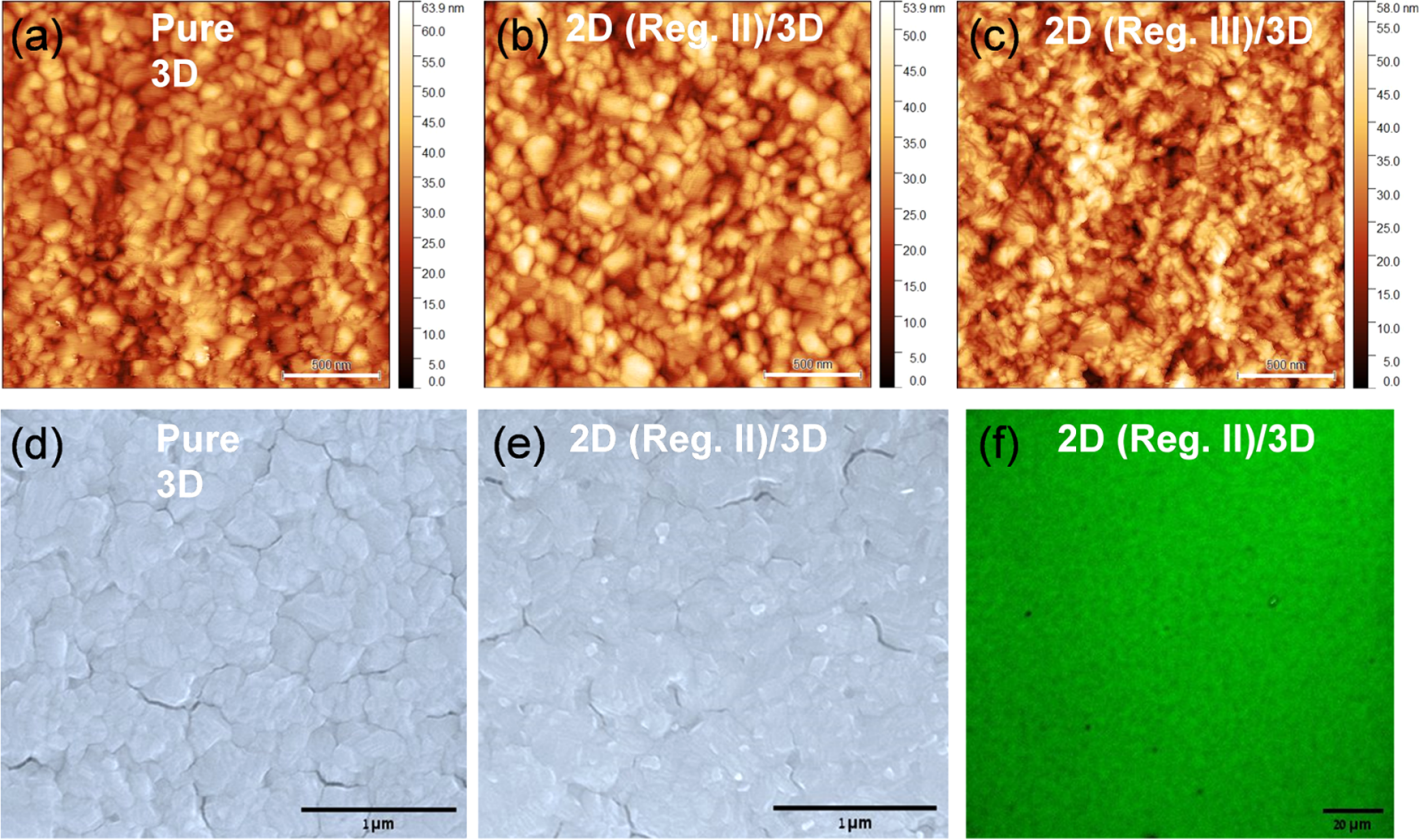
Surface morphology characterizations of the pure 3D and 2D-on-3D bilayer films by (a–c) AFM and (d, e) SEM scans. (f) Confocal PL map of 2D emission from 2D-on-3D bilayer films, excited with 488 nm laser. Regions of the PL intensity vs time profile during the 2D growth (Figure 1c) are marked within each image. Scale bars in AFM, SEM, and confocal PL images represent 500 nm, 1 μm, and 20 μm, respectively.

Uniform coverage of the surface cap is critical, and the more so, the thinner the capping layer is, which otherwise can result in partial shorts within the device, increasing recombination currents in a solar cell. Earlier efforts to realize uniform and ultrathin (<10 nm) overlayers with spin-coating in organic polymeric systems resulted in pinholes and/or partial surface coverage.^33,34^ Uniform surface coverage of ultrathin overlayers becomes even more critical on relatively rough surfaces, like those of polycrystalline HaP films with typical root-mean-square (rms) roughness of ca. 10–12 nm. Hence, it is critical to check if the surfaces, especially the ultrathin 2D-on-3D ones grown via controlled vapor-phase surface cation exchange, are continuous and cover the 3D HaP substrate conformally. Atomic force microscopy (AFM) topographic images (Figure 5a–c) were recorded for pristine and 2D-modified MAPbI_3_ thin films for which the 2D growth was terminated in regimes that yield ultrathin 2D capping layers, i.e., regimes II and III. Along with the top view SEM images of such 2D/3D bilayers shown in Figures 5d,e, and S9, they provide a view of the surface morphology. The root-mean-square roughness of the MAPbI_3_ thin films is ∼7.2 nm. The films exhibit a densely packed grain morphology with ca. 150–200 nm average grain size (Figure 5a). For the ultrathin 2D capping layers, i.e., for growth in reg. II, the surface morphology can hardly be distinguished from that of the pure 3D films, both in AFM (cf. Figure 5a with Figure 5b) and SEM (cf. Figure 5d with Figure 5e) images. However, for thicker 2D growth, i.e., in reg. III, a grain morphology change is discernible alongside a slight increase in rms surface roughness values from 7.3 to 8.4 nm, which is also visible in the SEM images shown in Figure S9a,b in the SI. Both AFM and SEM surface topographic images of the 2D/3D bilayers reveal that the surface morphology changes throughout on the 3D growth platforms after the 2D growth (compare Figure 5a,d with Figures 5c and S9a, respectively).

In contrast to spin-coating for 2D layer deposition, which is affected by the grain morphology (rms roughness) of the bottom substrate, the vapor-phase surface cation exchange is a very gradual 3D to 2D conversion process. The 3D to 2D conversion takes place homogeneously on the 3D surfaces exposed to 2D vapor molecules and is unlikely to be affected by the grain morphology of the 3D surface. The evidence for the homogeneity of 2D surface coverage can be seen by comparing the topographic AFM image in Figure 5c with the cross-sectional SEM images in Figure S5c, where the 2D overlayer films, ca. 10–20 nm in thicknesses uniformly covers the 3D HaP surface. Further, the conformity of the 2D layers is also seen in scanning transmission electron microscopy (STEM) bright-field images (Figure S9c,d) of 2D/3D bilayer cross sections where flat 2D grains (∼20 nm thick) can be seen uniformly covering the 3D surfaces. Note that the distinct contrast in the images relates to the different intensities of Bragg diffraction from 2D and 3D grains with different crystallinity. Hence, we presume that even for the ultrathin 2D layers grown in regime II, the surface conversion is uniform on the 3D surfaces, although we lack sensitivity to see a difference in surface morphology from that of the pristine 3D surface (Figure 5a,d compared with Figure 5b,e).

However, to get further information about the uniformity of 2D surface coverage on the 3D film, we studied the PL emission of the 2D layer by laser scanning confocal microscopy of the 2D/3D layers with 488 nm excitation. The PL emission map for the 2D emission band (500–550 nm) is shown in Figure 5f for the ultrathin 2D layers grown on top of the 3D surface (regime II in Figure 1c). The uniform green emission across the 140 × 140 μm^2^ area of the 2D emission map is consistent with spatial homogeneity, admittedly at a coarser scale, of the grown 2D phase on top of the 3D grains.

### 2.6 Ultrathin 2D Cap Suffices to Stabilize 3D HaP Layers

The vapor-phase growth of 2D on 3D HaP layers is topotactic as it yields 2D layers with strong chemical and structural correlation to the 3D HaP layers underneath. Also, because the 2D film grows within, and not on top of, the 3D HaP layers, the 2D layers are conformal, as was discussed in Section 2.5. Hence, it can be assumed that even for ultrathin (∼5 nm) 2D layers grown in regime II of the PL intensity vs time profile (Figure 1c), the 3D HaP underneath the 2D cap will be effectively passivated against environmental degradation, especially moisture.

**Figure 6:**
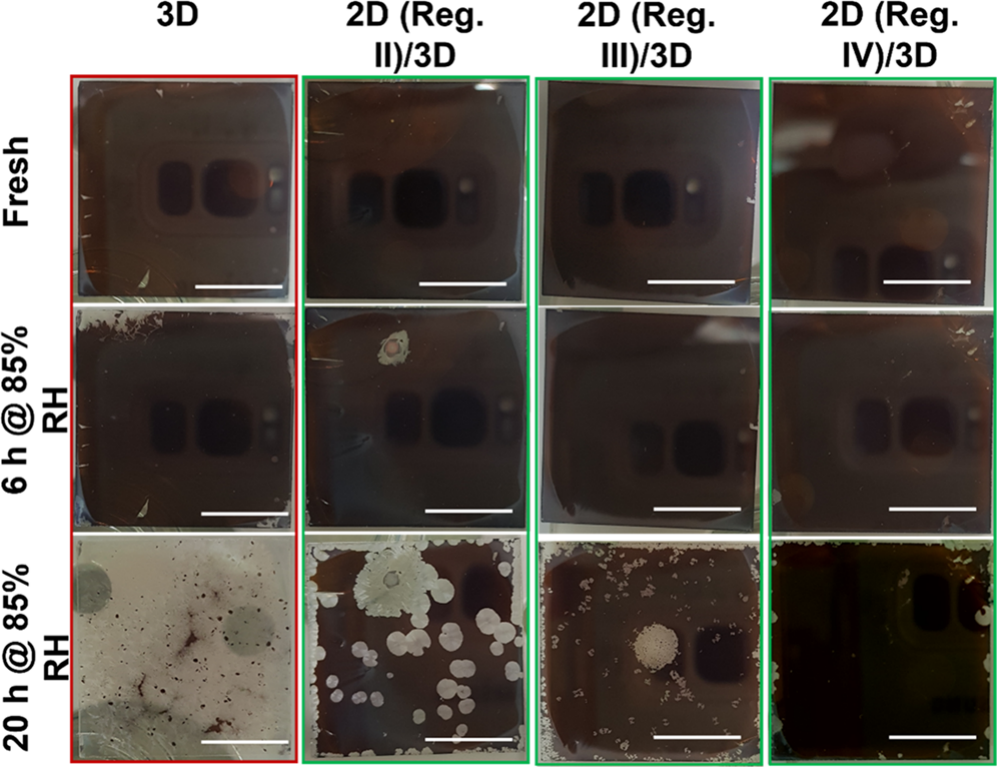
Stability test under ∼85% RH in the air at room-temperature conditions for pristine and 2D/3D bilayer perovskite films grown with different thicknesses. Each column represents a different growth regime defined in Figure 1c. first row: fresh, as-prepared 3D and 2D/3D layers; second row: after 6 h exposure to 85% RH; third row: after 20 h exposure to 85% RH. The scale bar represents 1 cm.

To evaluate the stability of 2D-coated 3D layers against moisture, we performed degradation tests on pristine 3D and 2D/3D samples by exposing them to high humidity conditions (>85% RH) in a custom-built chamber. The optical images of the corresponding films captured at different representative time intervals are shown in Figure 6. The untreated MAPbI_3_ films show a noticeable change in their appearance within a few hours of exposure to humidity; after ∼20 h exposure, the black perovskite phase has almost completely disappeared. However, all 3D films with a 2D capping on top do not show such severe damage (disappearance of the characteristic perovskite black phase).

For 2D layers on the 3D films that are relatively thick (>10 nm, grown till regime IV, and as deduced from XPS analysis), the MAPbI_3_ films do not show discoloration even after 20 h in the high humidity ambient. With thinner 2D layers, i.e., grown till regimes II and III, the 3D films are essentially unaffected by the humidity for at least 6 h. The films show noticeable discoloration only after 20 h of severe moisture exposure. The test indicates that even with ∼5 nm thick vapor-grown 2D layers, the 3D MAPbI_3_ should, with common encapsulation strategies,^35^ effectively be stable against typical ambient moisture levels of 40–60% RH.

The potential of the HaP materials to recover from external damage either intrinsically (self-heal) or by using other intentionally added components (repair) is as important as their ability to resist ambient-induced degradation, as all of these will add to the functional lifetimes of HaP-based optoelectronic devices, like solar cells.

MAPbI_3_ and other Pb-HaPs have shown a remarkable ability to self-heal after photodamage,^27,28^ and under mild pressure and at slightly elevated (above RT) temperatures.^36−39^ Thus, we studied the possible healing in 3D MAPbI_3_ thin film, capped with their structurally and chemically similar analogues, the 2D FPEA_2_PbI_4_ HaP.

**Figure 7:**
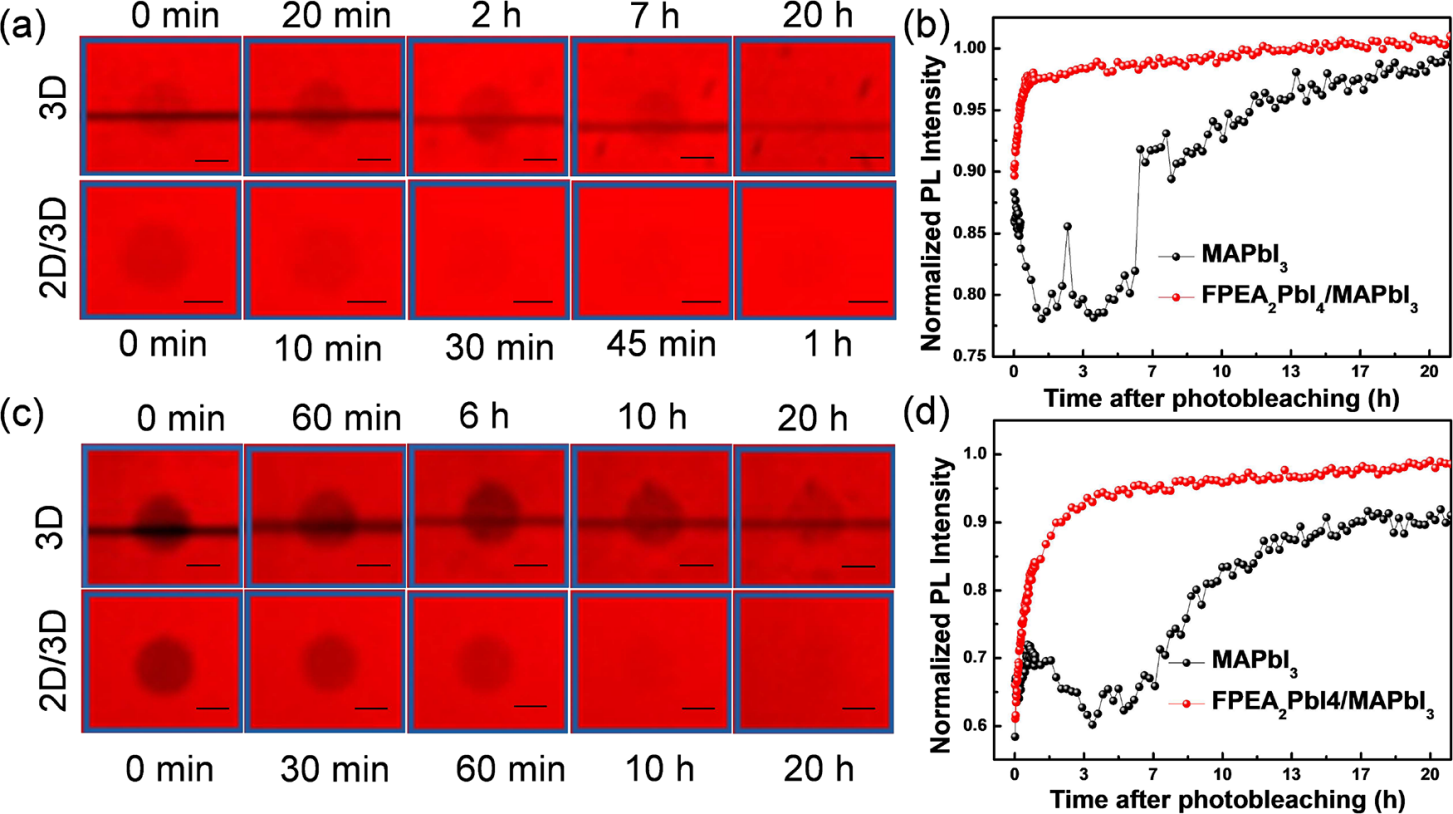
Maps and plots of PL (emission > 730 nm, i.e., from MAPI only), as a function of time after photodamage, of 3D MAPbI_3_ and ultrathin 2D-on-3D HaP films. (a, c) Time evolution of the PL emission maps of a photodamaged region (dark circular spot). (b, d) PL emission intensity vs time plots of the corresponding normalized PL intensity recovery from photodamage. 3D layers were photobleached with 488 nm laser pulses of different intensities: after the damage, the films retained ∼85% (i.e., mild, ∼15% photodamage; (a, b)) and ∼60% (i.e., moderate, ∼40% photodamage; (c, d)) of their initial PL intensity. For 2D/3D bilayers, the 2D growth was terminated in regime II of the PL intensity vs time profile, i.e., in Figure 1c.

To this end, we used fluorescence recovery after photobleaching (FRAP) on pure 3D and 2D/3D bilayer HaP films encapsulated with a polymer poly-iso-butylene (PIB) film. The encapsulation of the sample was necessary so as not to expose it, especially its photodamaged regions, to the ambient, as such exposure was shown to affect the PL recovery process negatively.^40^ A wavelength of 488 nm was used for both exciting and damaging the perovskite samples for these measurements. The damage on the perovskite regions was caused by scanning the confocal beam with an order of magnitude higher laser intensities (equivalent to few tens of solar illumination) than what was used for collecting the PL images (see Section S4 in the SI). We gauge the severity of the photodamage based on a percentage of immediate loss of PL intensity (measured with the low-intensity excitation) in the damaged ROIs normalized to the values in adjacent undamaged areas of the sample. Figures 7 and S10a,c show the PL intensity maps from a region of interest (ROI) on the 3D and 2D/3D perovskite samples as a function of time following a photodamage event (darker regions within ROI). Under mild and moderate photobleaching conditions, i.e., when the films retain at least about 60% of their initial PL intensity, the presence of the 2D layer improves PL recovery kinetics (Figure 7b,d). The 2D/3D layers recover ∼90% of their initial PL intensity just within 2 h and ∼100% after 10 h of the photodamage. In contrast, the pure 3D films (i.e., without any 2D capping) do not show significant PL recovery till 4–6 h of the initial damage, and only after ∼7 h, PL recovery starts with a steep slope, as shown in Figure 7b,d. Less striking but somewhat similar results are observed after more severe photobleaching, i.e., with only 0–30% of the initial PL remaining after the photo-bleach, as shown in Figure S10 in SI (see next paragraph).

The sudden increase of the PL intensity after ca. 7–8 h of the photodamage was noticeably absent for the 2D/3D bilayer samples, even in the severely photodamaged films (Figure S10). Instead, as shown in Figure S10, 20 h after 70% photodamage, the pure 3D HaP layers recovered more of the initial PL intensity than the 2D/3D bilayers. Note that in this case, the 2D/3D bilayers also showed faster PL recovery between 0 and 6 h after the photodamage, as was observed also for mild to moderate photodamages in these samples (cf. Figure 7).

A close investigation of the PL emission maps of the encapsulated 3D and 2D/3D HaP films measured after ca. 20–24 h of ambient exposure (Figure S11 in the SI) shows the formation of small needle-like entities with visibly no emission in the >730 nm wavelength range, on the 3D HaP surfaces. In contrast, such features were not observed for 2D/3D bilayer films. This indicates that the polymeric encapsulant, originally deposited to avoid unintended ambient interactions of the photodamaged perovskite surfaces (at least immediately after photodamage) with the ambient atmosphere, does not prevent ambient–HaP surface interactions after more prolonged exposure. Such unwarranted interactions with the ambient resulted in the observed dark features in the emission maps only for the case of pure 3D HaP films and may be related to the observed anomalous rise in their PL recovery after ca. 6–7 h of the photodamage (Figures 7b,d and S10b,d). Irrespective of this anomaly, the PL recovery after photodamage highlight: (1) an ultrathin 2D capping layer on a 3D HaP film can improve photodamage recovery at the 2D/3D interface for low to medium (in terms of loss of PL) photodamage, and (2) the anomalous PL increase/recovery after ca. 7–8 h of the photodamage can be due to the effect of the ambient (O_2_, H_2_O) on the PL properties of the (damaged) perovskite domains,^41^ which is absent if the films are capped with a 2D layer.

This latter observation is consistent with the results obtained in the stability test experiments performed on the 3D and 2D/3D HaP films (Figure 6) and demonstrates how ultrathin 2D coats are effective encapsulants for 3D HaPs. The improved self-healing kinetics of the 3D MAPbI_3_ surfaces, if capped with a 2D film measured by PL recovery is somewhat surprising. Below we give a possible chemical explanation for this.

When the 3D MAPbI_3_ surfaces are exposed to high-energy laser photons, the MAPbI_3_ lattice breaks down due to enthalpic instability toward decomposition.^42^ Methylammonium, MA^+^ can undergo photolysis, generating MA and a free proton (H^+^).
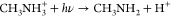
4

On free surfaces or in the absence of a suitable chemical environment that can retain gaseous products like methylamine, neutral MA will escape from the HaP lattice or diffuse away from the damaged region. The system will then not be able to revert to its original configuration, i.e., the MAPbI_3_ phase, even though the entropic driving force (to make Δ*G* negative) should favor its conversion from the binaries at equilibrium:
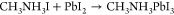
5

The PIB polymer on top of HaP films serves as a barrier, preventing or delaying the escape of methylamine (and other gaseous products that may form during photodamage). The PL map in Figure S11b suggests that for pure 3D MAPbI_3_, delaying is what happens (as humidity seems to affect the PL emission over 24 h of ambient storage). The improved self-healing kinetics of 3D photodamage if a 2D cap is present (for mild to moderate cases) suggest that the layered 2D structure helps prevent escape or out-diffusion (from the region of interest, ROI) of the gaseous products from the damaged region. Such can happen if the 2D layers cover the 3D HaP surfaces in a highly conformal manner and/or the organic barriers separating the [PbI_6_]^4–^ octahedra are able to contain all of the reaction products within the (2D/3D) HaP system. The nearly unaffected PL emission maps (Figure S11c,d) after 24 h of ambient storage imply that such is the case here as the result of diffusion of O_2_ or H_2_O from the external ambient is not observed with the 2D/3D structures.

Another possibility can arise if the methylamine (MA) binds to Pb^2+^ in the perovskite structure by coordination of the N lone pair with Pb^2+^. Such interaction (see eq 6) was shown earlier to be possible by density functional theory (DFT) calculations^40^ and was also demonstrated experimentally for methyl- and butyl-amines with Pb^2+^ in PbI_2_.^16,43^
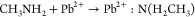
6

Naturally, having a 2D HaP overlayer can increase such binding as MA can now bind to the Pb^2+^ of the 2D layers, thereby reducing its loss after photodamage.

The free H^+^ in the perovskite lattice can reverse eqs 4 and 6 to reform MA^+^, and its (MA → MA^+^) rate will depend on the concentration of free H^+^ in the perovskite lattice, especially near the regions of the photodamage, i.e., at the 2D/3D interfaces. Such re-formation of the MA^+^ cations can then help photodamage healing of the perovskite structure (Figure 7). For 2D/3D HaP bilayers, the relative valence band positions of the 2D and 3D HaP layers (Figure 4c) pose an energetic barrier for the positively charged entities (like holes and H^+^) at the interface. This suggests that the free H^+^ generated within the HaP due to MA^+^ → MA decomposition (eq 4) can accumulate at the 3D/2D interface due to this energetic barrier restricting their drift into the 2D component. We assume that this accumulation of H^+^ makes the MA → MA^+^ conversion more accessible at the 2D/3D interface and could be a reason for the observed improved self-healing kinetics in contrast to bare 3D ones. Another rationale for this conjecture is the observation that bare 3D ones show anomalous recovery ca. 6–7 h after initial photodamage (Figures 7 and S10). Previously, we explained the abnormal PL recovery of bare 3D HaP photodamage by uncontrolled interactions of the perovskite lattice with the ambient species. Additionally, water diffusion, which earlier has been reported to enhance H^+^ migration within the perovskite lattice,^44^ could aid healing as improved H^+^ migration will help reverse the processes shown in eqs 4 and 6 to reform the MA^+^ ions. Moreover, water can itself act as a source for additional H^+^ ions within the perovskite lattice to shift the equilibrium in eqs 4 and 6 toward the left, i.e.,
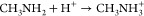
7
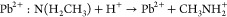
8

Our hypothesis that the accumulation of free H^+^ ions at the 2D/3D interface benefits healing should be further investigated, preferably using 2D HaP caps on MAPbI_3_ with band offsets favorable for H^+^ ion extraction. Such a study can help further (dis)prove our proposed mechanism for the improved self-healing of the 2D/3D HaP interfaces and also help find materials systems that can form even more electrically efficient and robust interfaces with the 3D HaPs.

## 3 Summary and Conclusions

2D-on-3D HaP bilayers with phase-pure 2D HaP are special as they form the ideal couple that fits the need for ambient stable and electronically transparent barrier with near-optimal 2D/3D interface for photovoltaic devices. Given that the 2D/3D heterointerfaces have enormous prospects for optoelectronic devices, having a facile route to control the 2D thicknesses down to ultrathin (<10 nm) regime with uniform surface coverage is essential, especially for larger-area devices.

We showed how controlled vapor-phase surface cation exchange produces such near-ideal 2D/3D heterointerfaces. *In situ* PL monitoring of the 3D surface reactions and 2D growth reveals a detailed picture of 3D to 2D conversion kinetics and enables tuning the 2D capping layer to a regime that yields a 2D thickness, which allows efficient electron tunneling, i.e., ≤∼5 nm. Uniform, conformal surface coverage was inferred from AFM, SEM, STEM, and confocal PL images of 2D/3D bilayer films. Furthermore, results from transient PL decay measurements show that energy-level mismatch between the 2D and 3D layers does not present a barrier for electronic transport if the 2D overlayers are ultrathin; otherwise, the band offsets between the 2D and 3D layers should be considered while using the heterojunction in devices. Most importantly, we show how even an ultrathin 2D capping on 3D HaP stabilizes the 3D surface in ambient humidity and also helps the healing of 3D photodamage in the HaP film caused by illumination up to the equivalent of a few tens of suns. We note that with the present experimental setup for the vapor-phase growth of ultrathin and phase-pure 2D layers, the ambient exposure of the 3D surfaces before/after the 2D growth cannot be completely avoided. Based on the results presented here, it should be possible to prepare ultrathin and phase-pure 2D layers on large-area perovskite substrates in a manner that will enable incorporating the process in device fabrication without any ambient exposure, a direction that will be explored in the future.

## 4 Experimental Section

### 4.1 3D MAPbI_3_ Preparation and 2D Growth

The 3D MAPbI_3_ thin films were deposited from a 1.4 M precursor solution prepared by mixing (∼1:1 ratio) methylammonium iodide (MAI, >99.9%, Greatcell Solar Materials) and lead iodide, PbI_2_ (99.99%, TCI Chemicals) in a 7:3 volume ratio of γ-butyrolactone and dimethyl sulfoxide, respectively. The 3D MAPbI_3_ thin films were deposited on cleaned and oxygen-plasma-treated substrates using an optimized two-stage spin coating recipe of first spinning at 1000 rpm for 10 s, followed by 4000 rpm for 30 s. 800 μL of toluene antisolvent was dripped on the spinning substrates at the 25th second of the second stage of spin-coating. The films were dried on a hotplate at 60 °C for 1 min, followed by at 100 °C for 5 min.^45−47^ The 2D growth on the 3D HaP surfaces was done by quickly transferring the MAPbI_3_ thin films into a custom-designed and built N_2_-filled chamber with an optically transparent viewport (Figure S1). The chamber was equipped with the necessary gas flow lines and flowmeters to regulate the flow of N_2_ and vapors of the 2D precursor (4-fluorophenethylamine, TCI) in and out of the growth chamber. Flowmeters, both for N_2_ dilution gas line and carrier gas into the FPEA bubbler, were set to optimum flow rates of 1.5 and 2 l/min, respectively, to ensure slow and uniform surface reaction of the MAPbI_3_ perovskite surfaces. For spin-coating of 2D layers, 4-fluoro-phenethylammonium iodide, FPEAI (Greatcell solar) was dissolved in anhydrous isopropanol (2 mg/mL) and spin-coated on 3D MAPbI_3_ surface at a speed of 4000 rpm for 30 s. All of the 2D/3D bilayer films were annealed at 80 °C for 4 min within the glovebox, prior to any characterizations performed on these layers.

### 4.2 *In Situ* PL Monitoring System

The photoluminescence measurements shown in this study were performed using a home-built optical setup (Figure S1) consisting of a diode pumped laser (Cobolt DPL -08), a laser clean-up filter Semrock −457.9 nm MaxLine (LL 457 nm), an ultrasteep long-pass edge filter Semrock-458 nm RazorEdge (RELP 458 nm), a 458 nm BragGrate Notch Filter (Notch 458 nm), a 10× objective with NA = 0.3, and a collimator package. The optics were built in reflection geometry, and RELP 458 nm acted as a mirror in excitation geometry and a spectral decoupler in reflection geometry. Slight angle tuning of RELP 458 was required. The 458 nm notch filter was inserted before the collimator to reject the excitation beam further. Standard optical alignment practices were followed in aligning the spectral beam with the collimator, and the spectrum was collected using a CCD-based spectrometer (Ocean Optics S2000).

### 4.3 Nanobeam Electron Diffraction Experiments

Thin (60–80 nm) cross-sectional lamellae of 2D/3D bilayers deposited on Si substrates were prepared with Helios 600 Dual Beam FIB-SEM instrument (Thermo Fisher Scientific) under conditions that minimize damage to the samples. Before their preparation using a focused ion beam (FIB), samples were coated with carbon (∼200 nm) and Pt (1.5 μm) protective coats. A ∼2 μm thick lamella was cut using focused Ga^+^ ions operated at an accelerating voltage of 30 kV and 2.8 nA beam current, which was then welded onto copper lift-out semigrid for further thinning down to 150 nm using lower beam currents of 300 to 50 pA. Final polishing of the lamellae to <100 nm thick was done at an even lower accelerating voltage of 5 kV and current of 50 pA. Scanning transmission electron microscopy (STEM) measurements were performed using a double aberration-corrected Themis-Z microscope (Thermo Fisher Scientific Electron Microscopy Solutions) at an accelerating voltage of 200 kV and an electron probe convergence angle of 0.2 mrad. For scanning beam electron diffraction experiments, the electron probe was typically defocused by 5–10 μm to increase the real space beam diameter and minimize the damage to the sample. This resulted in a probe size of a few tens of nanometers which scanned through the sample with 1 ms exposure per probe position on the sample. The primary beam current was between 1 and 4 pA. An electron microscope pixel array detector (EMPAD) was used to record high-quality electron diffraction patterns with high frames per second readout speeds.

### 4.4 XPS and UPS Characterization

XPS and UPS measurements were performed with a Kratos AXIS ULTRA system equipped with a concentric hemispherical analyzer for detecting the photo-excited electrons. A monochromatic Al-Kα X-ray source (*hν* = 1486.6 eV) at 75 W and detection pass energies ranging between 20 and 80 eV were used for XPS measurements. UPS was measured with a helium discharge lamp, using He I (21.22 eV) and He II (40.8 eV) radiation lines. The total energy resolution for the measurements was less than 100 meV, determined from the Fermi edge of the Au reference sample. All UPS spectra were recorded with −10 V bias applied to the sample to detect secondary electron photoemission cutoff at low kinetic energies.

### 4.5 Photovoltaic Device Fabrication

Indium-doped tin oxide (ITO) glass substrates were cleaned by ultrasonication sequentially in detergent, acetone, isopropanol, and deionized water for 15 min each. The clean ITO substrates were dried under nitrogen flow and treated with O_2_-plasma for 3 min prior to the deposition of the electron transporting layer (ETL). SnO_2_ (ETL, ∼30 nm) was deposited by spin coating a SnO_2_ nanoparticles dispersion (7.5 wt %) in water at 3000 rpm for the 30 s. The SnO_2_ layer was then annealed on a hotplate at 180 °C for 1 h. For the deposition of 3D HaP layers on SnO_2_/ITO substrates and consequent vapor-phase 2D growth, a similar protocol was followed as described above in 3D MAPbI_3_ preparation and 2D growth. The spiro-OMeTAD HTL layer was deposited following a recipe, similar to that described in the literature.^45,48^ Finally, Ag electrodes (∼3 mm diameter) were deposited on the as-prepared PV stack by thermal evaporation under high vacuum conditions (10^–6^ mbar).

## Data Availability

All of the data supporting this study’s findings are included in the article and the Supporting Information.

## Supporting Information Available

Additional information on the experimental setup, XPS and UPS analysis, optical, electron microscopy, and device characterizations on 2D/3D HaP bilayers (PDF)

## References

[ref1] Jena A. K.; Kulkarni A.; Miyasaka T. Halide Perovskite Photovoltaics: Background, Status, and Future Prospects. Chem. Rev. 2019, 119, 3036–3103. 10.1021/acs.chemrev.8b00539. 30821144

[ref2] Gao Y.; Pan Y.; Zhou F.; Niu G.; Yan C. Lead-Free Halide Perovskites: A Review of the Structure-Property Relationship and Applications in Light Emitting Devices and Radiation Detectors. J. Mater. Chem. A 2021, 9, 11931–11943. 10.1039/d1ta01737c.

[ref3] Green M. A.; Dunlop E. D.; Siefer G.; Yoshita M.; Kopidakis N.; Bothe K.; Hao X. Solar Cell Efficiency Tables (Version 61). Prog. Photovoltaics: Res. Appl. 2023, 31, 3–16. 10.1002/pip.3646.

[ref4] Correa-Baena J. P.; Saliba M.; Buonassisi T.; Grätzel M.; Abate A.; Tress W.; Hagfeldt A. Promises and Challenges of Perovskite Solar Cells. Science 2017, 358, 739–744. 10.1126/science.aam6323. 29123060

[ref5] Stoumpos C. C.; Cao D. H.; Clark D. J.; Young J.; Rondinelli J. M.; Jang J. I.; Hupp J. T.; Kanatzidis M. G. Ruddlesden-Popper Hybrid Lead Iodide Perovskite 2D Homologous Semiconductors. Chem. Mater. 2016, 28, 2852–2867. 10.1021/acs.chemmater.6b00847.

[ref6] Cao D. H.; Stoumpos C. C.; Farha O. K.; Hupp J. T.; Kanatzidis M. G. 2D Homologous Perovskites as Light-Absorbing Materials for Solar Cell Applications. J. Am. Chem. Soc. 2015, 137, 7843–7850. 10.1021/jacs.5b03796. 26020457

[ref7] Zhang X.; Wu G.; Yang S.; Fu W.; Zhang Z.; Chen C.; Liu W.; Yan J.; Yang W.; Chen H. Vertically Oriented 2D Layered Perovskite Solar Cells with Enhanced Efficiency and Good Stability. Small 2017, 13, 1700611 10.1002/smll.201700611. 28692766

[ref8] Straus D. B.; Kagan C. R. Electrons, Excitons, and Phonons in Two-Dimensional Hybrid Perovskites: Connecting Structural, Optical, and Electronic Properties. J. Phys. Chem. Lett. 2018, 9, 1434–1447. 10.1021/acs.jpclett.8b00201. 29481089

[ref9] Liu Y.; Akin S.; Pan L.; Uchida R.; Arora N.; Milić J. V.; Hinderhofer A.; Schreiber F.; Uhl A. R.; Zakeeruddin S. M.; Hagfeldt A.; Ibrahim Dar M.; Grätzel M. Ultrahydrophobic 3D/2D Fluoroarene Bilayer-Based Water-Resistant Perovskite Solar Cells with Efficiencies Exceeding 22%. Sci. Adv. 2019, 5, eaaw2543 10.1126/sciadv.aaw2543. 31187060 PMC6555633

[ref10] Sutanto A. A.; Caprioglio P.; Drigo N.; Hofstetter Y. J.; Garcia-Benito I.; Queloz V. I. E.; Neher D.; Nazeeruddin M. K.; Stolterfoht M.; Vaynzof Y.; Grancini G. 2D/3D Perovskite Engineering Eliminates Interfacial Recombination Losses in Hybrid Perovskite Solar Cells. Chem 2021, 7, 1903–1916. 10.1016/j.chempr.2021.04.002.

[ref11] Zhou Q.; Liang L.; Hu J.; Cao B.; Yang L.; Wu T.; Li X.; Zhang B.; Gao P. High-Performance Perovskite Solar Cells with Enhanced Environmental Stability Based on a (p-FC6H4C2H4NH3)2[PbI4] Capping Layer. Adv. Energy Mater. 2019, 9, 1802595 10.1002/aenm.201802595.

[ref12] Lv Y.; Song X.; Yin Y.; Feng Y.; Ma H.; Hao C.; Jin S.; Shi Y. Hexylammonium Iodide Derived Two-Dimensional Perovskite as Interfacial Passivation Layer in Efficient Two-Dimensional/Three-Dimensional Perovskite Solar Cells. ACS Appl. Mater. Interfaces 2020, 12, 698–705. 10.1021/acsami.9b17930. 31815408

[ref13] Chen J.; Seo J. Y.; Park N. G. Simultaneous Improvement of Photovoltaic Performance and Stability by In Situ Formation of 2D Perovskite at (FAPbI3)0.88(CsPbBr3)0.12/CuSCN Interface. Adv. Energy Mater. 2018, 8, 1702714 10.1002/aenm.201702714.

[ref14] Tan S.; Huang T.; Yavuz I.; Wang R.; Weber M. H.; Zhao Y.; Abdelsamie M.; Liao M. E.; Wang H. C.; Huynh K.; Wei K. H.; Xue J.; Babbe F.; Goorsky M. S.; Lee J. W.; Sutter-Fella C. M.; Yang Y. Surface Reconstruction of Halide Perovskites during Post-Treatment. J. Am. Chem. Soc. 2021, 143, 6781–6786. 10.1021/jacs.1c00757. 33915050

[ref15] Roose B.; Dey K.; Chiang Y. H.; Friend R. H.; Stranks S. D. Critical Assessment of the Use of Excess Lead Iodide in Lead Halide Perovskite Solar Cells. J. Phys. Chem. Lett. 2020, 11, 6505–6512. 10.1021/acs.jpclett.0c01820. 32693601

[ref16] Liu Z.; Meng K.; Wang X.; Qiao Z.; Xu Q.; Li S.; Cheng L.; Li Z.; Chen G. In Situ Observation of Vapor-Assisted 2D-3D Heterostructure Formation for Stable and Efficient Perovskite Solar Cells. Nano Lett. 2020, 20, 1296–1304. 10.1021/acs.nanolett.9b04759. 31986053

[ref17] Sutanto A. A.; Drigo N.; Queloz V. I. E.; Garcia-Benito I.; Kirmani A. R.; Richter L. J.; Schouwink P. A.; Cho K. T.; Paek S.; Nazeeruddin M. K.; Grancini G. Dynamical Evolution of the 2D/3D Interface: A Hidden Driver behind Perovskite Solar Cell Instability. J. Mater. Chem. A 2020, 8, 2343–2348. 10.1039/c9ta12489f.

[ref18] Vázquez-Cárdenas R.; Rodríguez-Romero J.; Echeverría-Arrondo C.; Sanchez-Diaz J.; Chirvony V. S.; Martínez-Pastor J. P.; Díaz-Leyva P.; Reyes-Gómez J.; Zarazua I.; Mora-Seró I. Suppressing the Formation of High N-Phase and 3D Perovskites in the Fabrication of Ruddlesden-Popper Perovskite Thin Films by Bulky Organic Cation Engineering. Chem. Mater. 2022, 34, 3076–3088. 10.1021/acs.chemmater.1c04135.

[ref19] Niu T.; Lu J.; Jia X.; Xu Z.; Tang M. C.; Barrit D.; Yuan N.; Ding J.; Zhang X.; Fan Y.; Luo T.; Zhang Y.; Smilgies D. M.; Liu Z.; Amassian A.; Jin S.; Zhao K.; Liu S. Interfacial Engineering at the 2D/3D Heterojunction for High-Performance Perovskite Solar Cells. Nano Lett. 2019, 19, 7181–7190. 10.1021/acs.nanolett.9b02781. 31479275

[ref20] Gharibzadeh S.; Abdollahi Nejand B.; Jakoby M.; Abzieher T.; Hauschild D.; Moghadamzadeh S.; Schwenzer J. A.; Brenner P.; Schmager R.; Haghighirad A. A.; Weinhardt L.; Lemmer U.; Richards B. S.; Howard I. A.; Paetzold U. W. Record Open-Circuit Voltage Wide-Bandgap Perovskite Solar Cells Utilizing 2D/3D Perovskite Heterostructure. Adv. Energy Mater. 2019, 9, 1803699 10.1002/aenm.201803699.

[ref21] Chen H.; Teale S.; Chen B.; Hou Y.; Grater L.; Zhu T.; Bertens K.; Park S. M.; Atapattu H. R.; Gao Y.; Wei M.; Johnston A. K.; Zhou Q.; Xu K.; Yu D.; Han C.; Cui T.; Jung E. H.; Zhou C.; Zhou W.; Proppe A. H.; Hoogland S.; Laquai F.; Filleter T.; Graham K. R.; Ning Z.; Sargent E. H. Quantum-Size-Tuned Heterostructures Enable Efficient and Stable Inverted Perovskite Solar Cells. Nat. Photonics 2022, 16, 352–358. 10.1038/s41566-022-00985-1.

[ref22] Xing J.; Zhao Y.; Askerka M.; Quan L. N.; Gong X.; Zhao W.; Zhao J.; Tan H.; Long G.; Gao L.; Yang Z.; Voznyy O.; Tang J.; Lu Z. H.; Xiong Q.; Sargent E. H. Color-Stable Highly Luminescent Sky-Blue Perovskite Light-Emitting Diodes. Nat. Commun. 2018, 9, 3541 10.1038/s41467-018-05909-8. 30166537 PMC6117319

[ref23] Cao Q.; Li P.; Chen W.; Zang S.; Han L.; Zhang Y.; Song Y. Two-Dimensional Perovskites: Impacts of Species, Components, and Properties of Organic Spacers on Solar Cells. Nano Today 2022, 43, 101394 10.1016/j.nantod.2022.101394.

[ref24] Yoo J. J.; Seo G.; Chua M. R.; Park T. G.; Lu Y.; Rotermund F.; Kim Y. K.; Moon C. S.; Jeon N. J.; Correa-Baena J. P.; Bulović V.; Shin S. S.; Bawendi M. G.; Seo J. Efficient Perovskite Solar Cells via Improved Carrier Management. Nature 2021, 590, 587–593. 10.1038/s41586-021-03285-w. 33627807

[ref25] Wu Y.; Wang Q.; Chen Y.; Qiu W.; Peng Q. Stable Perovskite Solar Cells with 25.17% Efficiency Enabled by Improving Crystallization and Passivating Defects Synergistically. Energy Environ. Sci. 2022, 15, 4700–4709. 10.1039/d2ee02277j.

[ref26] Song Y.; Zhang C.; Liu W.; Li X.; Long H.; Wang K.; Wang B.; Lu P. High-Efficiency Energy Transfer in Perovskite Heterostructures. Opt. Express 2018, 26, 18448 10.1364/oe.26.018448. 30114024

[ref27] Yao Q.; Xue Q.; Li Z.; Zhang K.; Zhang T.; Li N.; Yang S.; Brabec C. J.; Yip H. L.; Cao Y. Graded 2D/3D Perovskite Heterostructure for Efficient and Operationally Stable MA-Free Perovskite Solar Cells. Adv. Mater. 2020, 32, 2000571 10.1002/adma.202000571. 32449209

[ref28] Garai R.; Gupta R. K.; Hossain M.; Iyer P. K. Surface Recrystallized Stable 2D-3D Graded Perovskite Solar Cells for Efficiency beyond 21%. J. Mater. Chem. A 2021, 9, 26069–26076. 10.1039/d1ta06901b.

[ref29] Kumar S.; Houben L.; Rechav K.; Cahen D. Halide Perovskite Dynamics at Work: Large Cations at 2D-on-3D Interfaces Are Mobile. Proc. Natl. Acad. Sci. U.S.A. 2022, 119, e2114740119 10.1073/pnas.2114740119. 35239436 PMC8915997

[ref30] Nguyen Q. V.; Frisbie C. D. Hopping Conductance in Molecular Wires Exhibits a Large Heavy-Atom Kinetic Isotope Effect. J. Am. Chem. Soc. 2021, 143, 2638–2643. 10.1021/jacs.0c12244. 33587628

[ref31] Gélvez-Rueda M. C.; Fridriksson M. B.; Dubey R. K.; Jager W. F.; van der Stam W.; Grozema F. C. Overcoming the Exciton Binding Energy in Two-Dimensional Perovskite Nanoplatelets by Attachment of Conjugated Organic Chromophores. Nat. Commun. 2020, 11, 1901 10.1038/s41467-020-15869-7. 32312981 PMC7171160

[ref32] Blancon J. C.; Stier A. V.; Tsai H.; Nie W.; Stoumpos C. C.; Traoré B.; Pedesseau L.; Kepenekian M.; Katsutani F.; Noe G. T.; Kono J.; Tretiak S.; Crooker S. A.; Katan C.; Kanatzidis M. G.; Crochet J. J.; Even J.; Mohite A. D. Scaling Law for Excitons in 2D Perovskite Quantum Wells. Nat. Commun. 2018, 9, 2254 10.1038/s41467-018-04659-x. 29884900 PMC5993799

[ref33] Hawash Z.; Ono L. K.; Raga S. R.; Lee M. V.; Qi Y. Air-Exposure Induced Dopant Redistribution and Energy Level Shifts in Spin-Coated Spiro-Meotad Films. Chem. Mater. 2015, 27, 562–569. 10.1021/cm504022q.

[ref34] Semaltianos N. G. Spin-Coated PMMA Films. Microelectron. J. 2007, 38, 754–761. 10.1016/j.mejo.2007.04.019.

[ref35] Mendez L R. D.; Breen B. N.; Cahen D. Lead Sequestration from Halide Perovskite Solar Cells with a Low-Cost Thiol-Containing Encapsulant. ACS Appl. Mater. Interfaces 2022, 14, 29766–29772. 10.1021/acsami.2c05074. 35735116 PMC9264311

[ref36] Ceratti D. R.; Rakita Y.; Cremonesi L.; Tenne R.; Kalchenko V.; Elbaum M.; Oron D.; Potenza M. A. C.; Hodes G.; Cahen D. Self-Healing Inside APbBr3 Halide Perovskite Crystals. Adv. Mater. 2018, 30, 1706273 10.1002/adma.201706273. 29328524

[ref37] Ceratti D. R.; Tenne R.; Bartezzaghi A.; Cremonesi L.; Segev L.; Kalchenko V.; Oron D.; Alberto M.; Potenza C.; Hodes G.; Cahen D. Self-Healing and Light-Soaking in MAPbI_3_: The Effect of H2O. Adv. Mater. 2022, 34, 2110239 10.1002/adma.202110239. 35731235

[ref38] Al-Handawi M. B.; Dushaq G.; Commins P.; Karothu D. P.; Rasras M.; Catalano L.; Naumov P. Autonomous Reconstitution of Fractured Hybrid Perovskite Single Crystals. Adv. Mater. 2022, 34, 2109374 10.1002/adma.202109374. 35234306

[ref39] Yadavalli S. K.; Dai Z.; Zhou H.; Zhou Y.; Padture N. P. Facile Healing of Cracks in Organic–Inorganic Halide Perovskite Thin Films. Acta Mater. 2020, 187, 112–121. 10.1016/j.actamat.2020.01.040.

[ref40] Ceratti D. R.; Cohen A. V.; Tenne R.; Rakita Y.; Snarski L.; Jasti N. P.; Cremonesi L.; Cohen R.; Weitman M.; Bendikov T.; Kalchenko V.; Elbaum M.; Potenza M. A. C.; Kronik L.; Hodes G.; Cahen D. The Pursuit of Stability in Halide Perovskites: The Monovalent Cation and the Key for Surface and Bulk Self-healing. Mater. Horiz. 2021, 8, 1570–1586. 10.1039/d1mh00006c. 34846465

[ref41] Song Z.; Shrestha N.; Watthage S. C.; Liyanage G. K.; Almutawah Z. S.; Ahangharnejhad R. H.; Phillips A. B.; Ellingson R. J.; Heben M. J. Impact of Moisture on Photoexcited Charge Carrier Dynamics in Methylammonium Lead Halide Perovskites. J. Phys. Chem. Lett. 2018, 9, 6312–6320. 10.1021/acs.jpclett.8b02595. 30336064

[ref42] Ciccioli A.; Latini A. Thermodynamics and the Intrinsic Stability of Lead Halide Perovskites CH3NH3PbX3. J. Phys. Chem. Lett. 2018, 9, 3756–3765. 10.1021/acs.jpclett.8b00463. 29901394

[ref43] Chen H.; Ye F.; Tang W.; He J.; Yin M.; Wang Y.; Xie F.; Bi E.; Yang X.; Grätzel M.; Han L. A solvent- and vacuum-free route to large-area perovskite films for efficient solar modules. Nature 2017, 550, 92–95. 10.1038/nature23877. 28869967

[ref44] Ceratti D. R.; Zohar A.; Kozlov R.; Dong H.; Uraltsev G.; Girshevitz O.; Pinkas I.; Avram L.; Hodes G.; Cahen D. Eppur Si Muove: Proton Diffusion in Halide Perovskite Single Crystals. Adv. Mater. 2020, 32, 2002467 10.1002/adma.202002467. 33048452

[ref45] Kumar S.; Choi Y.; Kang S. H.; Oh N. K.; Lee J.; Seo J.; Jeong M.; Kwon H. W.; II Seok S.; Yang C.; Park H. Multifaceted Role of a Dibutylhydroxytoluene Processing Additive in Enhancing the Efficiency and Stability of Planar Perovskite Solar Cells. ACS Appl. Mater. Interfaces 2019, 11, 38828–38837. 10.1021/acsami.9b14423. 31556588

[ref46] Mohanty I.; Mangal S.; Jana S.; Singh U. P. Post Annealing Effects of Perovskite (CH3NH3PbI3) Thin Films for Solar Cell Applications. In AIP Conference Proceedings; AIP Publishing, 2021, 2369 (September); p 020046. 10.1063/5.0061270.

[ref47] Chen L. C.; Chen C. C.; Chen J. C.; Wu C. G. Annealing Effects on High-Performance CH3NH3PbI3 Perovskite Solar Cells Prepared by Solution-Process. Sol. Energy 2015, 122, 1047–1051. 10.1016/j.solener.2015.10.019.

[ref48] Saliba M.; Correa-Baena J. P.; Wolff C. M.; Stolterfoht M.; Phung N.; Albrecht S.; Neher D.; Abate A. How to Make over 20% Efficient Perovskite Solar Cells in Regular (n-i-p) and Inverted (p-i-n) Architectures. Chem. Mater. 2018, 30, 4193–4201. 10.1021/acs.chemmater.8b00136.

