# Supplementary material for: Topotactic, Vapor-Phase, In Situ Monitored Formation of Ultrathin, Phase-Pure 2D-on-3D Halide Perovskite Surfaces
Source: ACS Appl Mater Interfaces. 2023 May 3;15(19):23908–21. doi: 10.1021/acsami.3c01881 (PMC10197072; doi:10.1021/acsami.3c01881)
Supplement: Supplementary file 1 — am3c01881_si_001.pdf [file am3c01881_si_001.pdf]

Supplementary Information

Topotactic, Vapor-Phase, *in situ* Monitored Formation of *Ultra-thin*, Phase-Pure 2D-on-3D Halide Perovskite Surfaces

Sujit Kumar[1,2 *] Vinayaka H. Damle[2], Tatyana Bendikov[3], Anat Itzhak[2], Michael Elbaum[4], Katya Rechav[3], Lothar Houben[3], Yaakov Tischler[2], David Cahen[1,2*]

[1]Dept. of Mol. Chem. & Mater. Science, Weizmann Inst. of Science, Rehovot-7610001 Israel

[2]Bar-Ilan Inst. for Adv. Mater. & Nanotech. & Dept. of Chem., Bar-Ilan Univ., Ramat Gan-5290002, Israel

[3]Dept. of Chem. Research Support, Weizmann Institute of Science, Rehovot-7610001, Israel

[4] Dept. of Chem. Biol. Physics, Weizmann Institute of Science, Rehovot-7610001, Israel

*Authors for correspondence: sujit.kumar@weizmann.ac.il; david.cahen@weizmann.ac.il

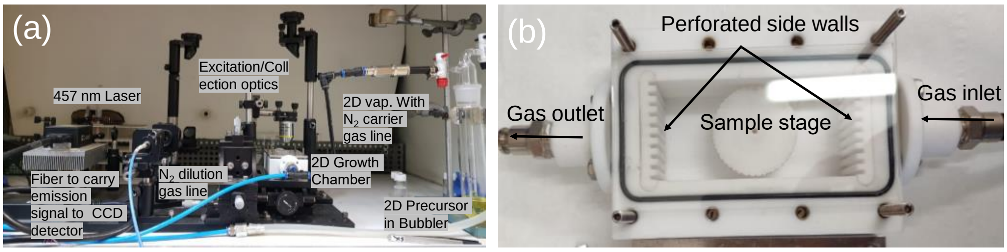

*Figure S1: (**a**) Custom-built 2D-on-3D HaP growth chamber along with optical excitation and PL collection optics (**b**) Top view of the reaction chamber for 2D growth*

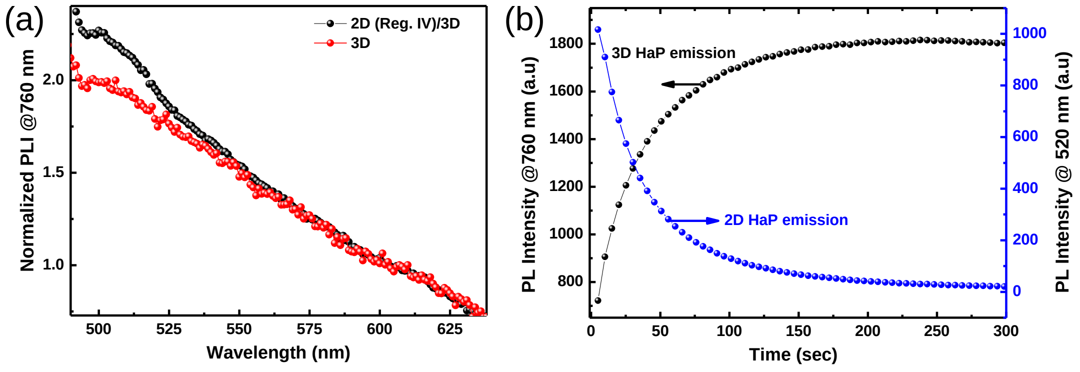

*Figure S2: (**a**) PL Excitation Spectra of $MAPbI_3$ thin films with and without a 2D $FPEA_2PbI_4$ perovskite layer (regime IV, Fig. 1c). The spectra are normalized with respect to the PL intensity at ~630 nm, i.e., well away from the 2D $FPEA_2PbI_4$ absorption region (450-515 nm). The increased PL intensity in the 490-515 nm wavelength region is ascribed to energy and/or electron transfer from the 2D to 3D perovskite. (**b**) Time evolution of 2D (520 nm) and 3D (760 nm) PL intensity after the photoexcitation for a 2D/3D bilayer film with thick 2D overlayer grown in regime IV. PL intensities of 2D and 3D emission peaks were recorded immediately after exciting the sample with 457 nm laser excitation.*

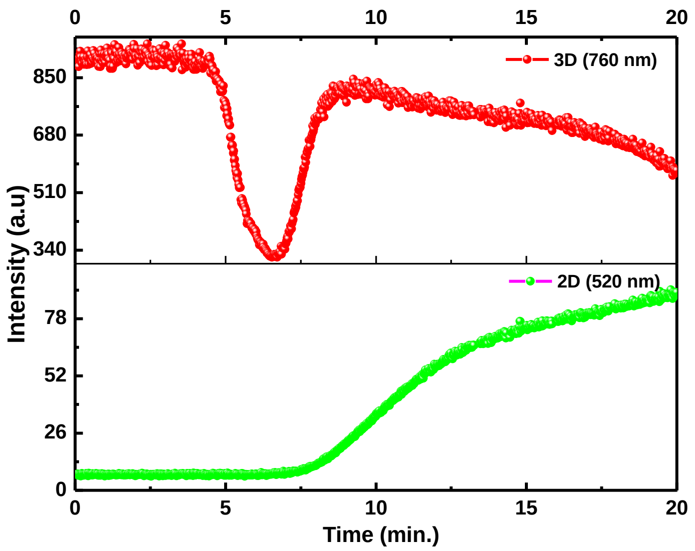

Figure S3: Evolution of PL intensity as a function of exposure time of the 3D $MAPbI_3$ HaP films to N_2 carrier gas, with vapors of 2D FPEA molecules, measured using the custom-built system for 2D growth and optics for in situ PL measurements, shown in Fig. S1.

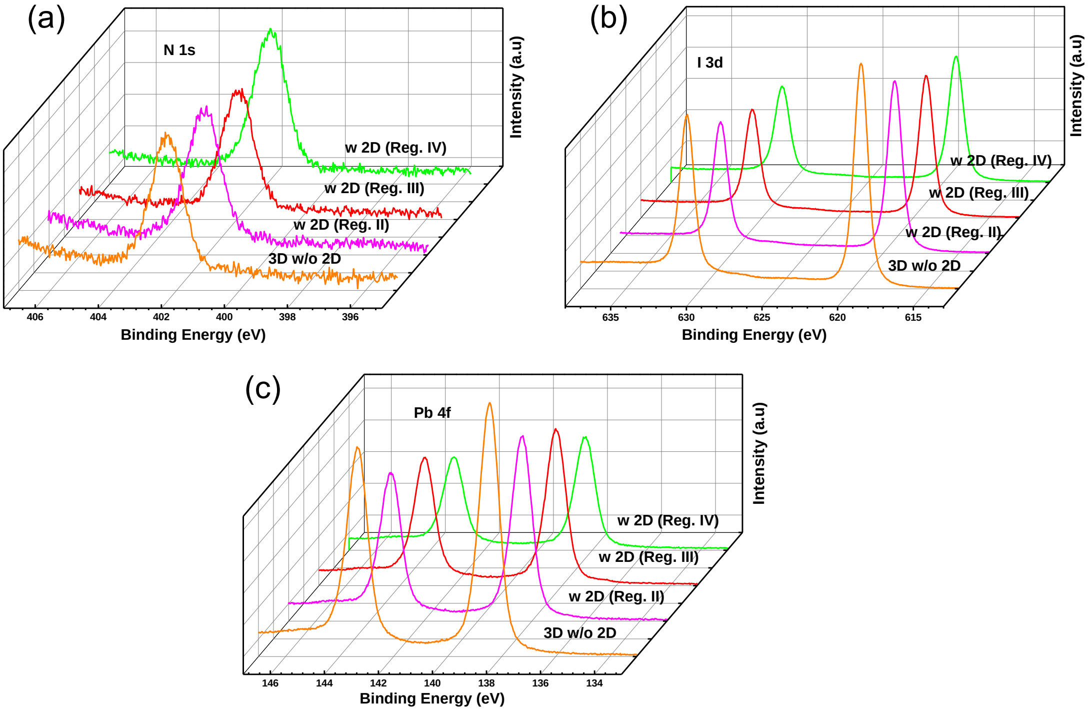

*Figure S4: High-resolution XPS spectra for (**a**) N 1s, (**b**) I 3d, and (**c**) Pb 4f electrons for 3D and 2D-on-3D perovskite bilayer films. Reg. II, III, or IV refer to the regime of the PL intensity vs. time profile in which 2D growth on the 3D HaP film was terminated (cf. Fig. 1c). Notably, among other features, the N 1s peak intensity increases with increasing 2D thickness, which fits with the extra nitrogen atoms present in the Fluoro-phenethyl ammonium cation of the 2D capping layer.*

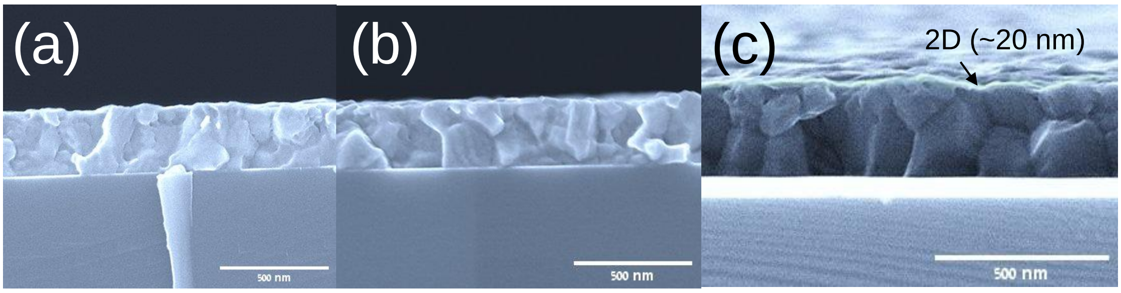

*Figure S5: Cross-sectional SEM images of 2D/3D HaP bilayers with 2D growth terminated in regime II- (**a**), III-(**b**), IV (**c**) of the PL intensity vs. time profile, shown in Fig. 1c. In (**a**) and (**b**) the 2D is indistinguishable from the 3D $MAPbI_3$ layers on which it grows. Only with the relatively thicker 2D, grown till regime IV (**c**), the 2D cap can faintly be distinguished from the 3D film, represented by the false coloring of the 2D overlayer.*

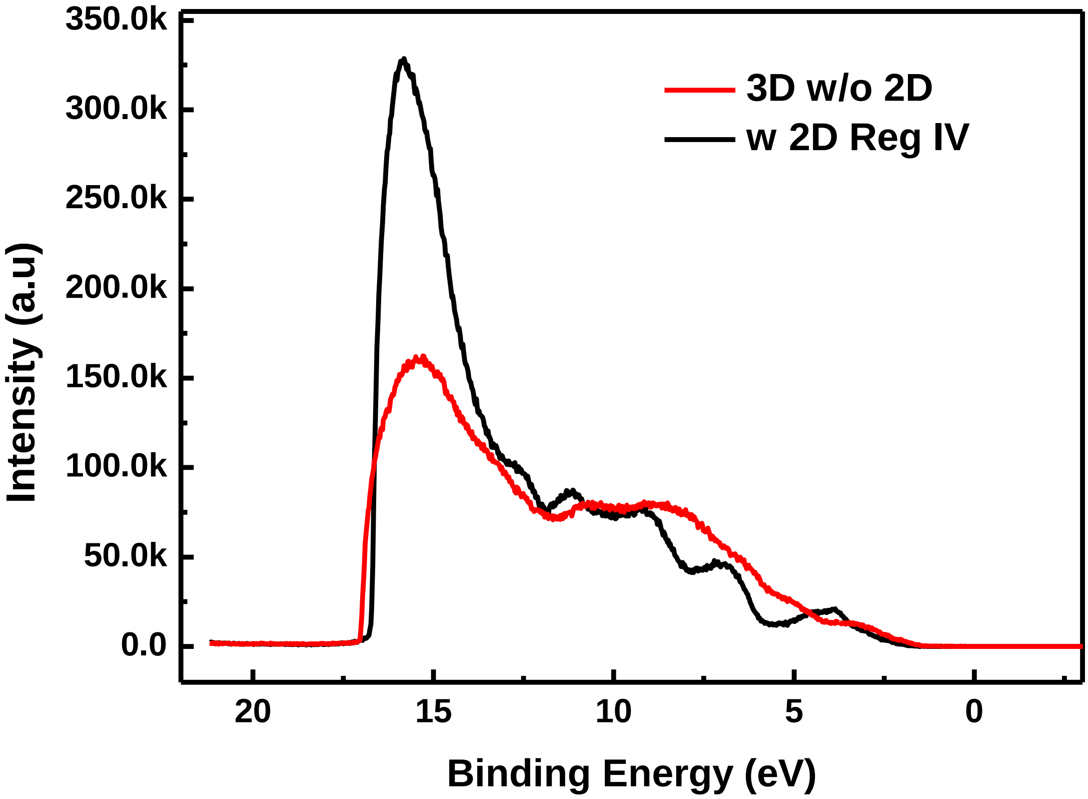

Figure S6: Full UPS spectra for 3D and 2D/3D bilayers. 2D HaP grown till regime IV of the PL intensity vs. time profile shown in Fig. 1c of the main text. For analysis, see section S3 below.

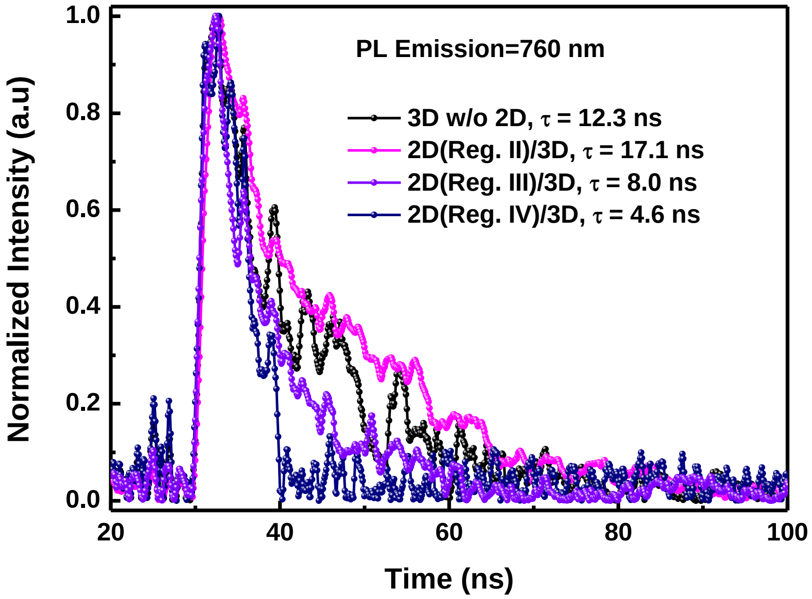

Figure S7: Transient PL decay characteristics of 3D and 2D/3D bilayers. The perovskite layers were excited with a 450 nm picosecond pulsed diode laser source, and decay of the photoexcited carriers was recorded using a photomultiplier tube.

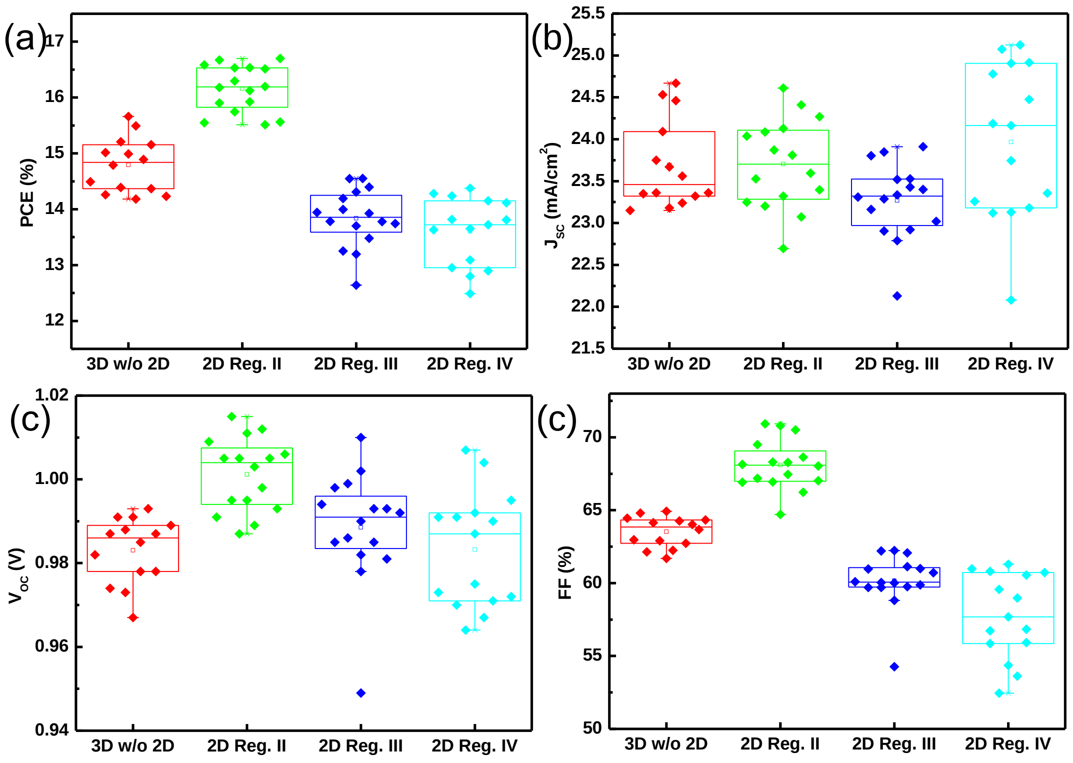

Fig. S8: Statistical distribution of PV parameters− PCE (a), J_{SC} (b), V_{OC} (c), and FF (d) for ~ 15 PV devices fabricated with 3D and vapor-phase grown 2D-on-3D HaP layers.

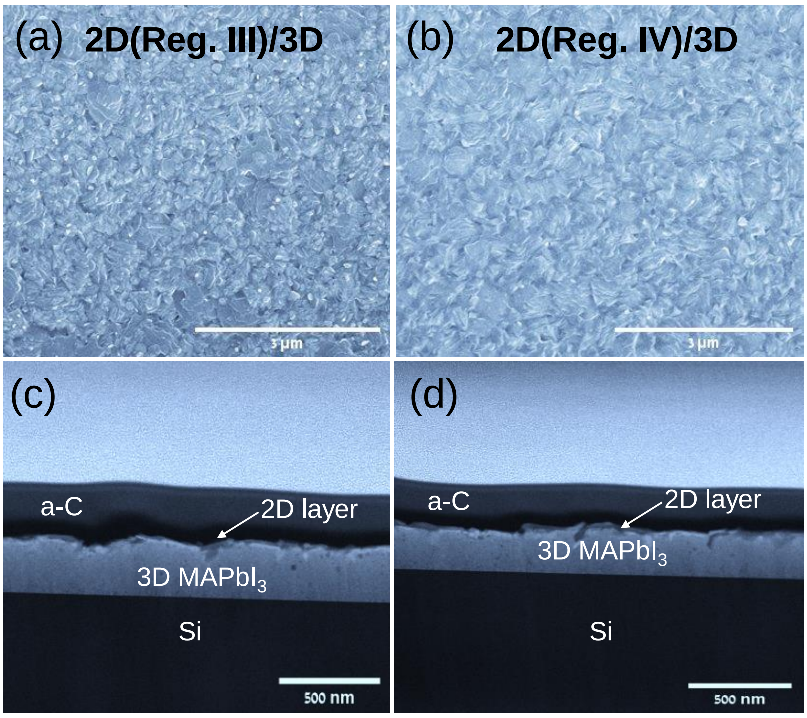

*Figure S9: (a), (b) Top view SEM images for 2D/3D bilayer films, with exposure until (including) regimes III (**a**) and IV (**b**), from Fig 1c, where the latter has a relatively thick, >10 nm, 2D layer. (**c**) and (**d**) STEM bright-field images (at different locations) of 2D/3D bilayer films with relatively thick 2D caps (regime IV) deposited on Si substrates. Amorphous Carbon (a-C) was deposited as a protective layer for FIB preparation of the cross-sections. Scale bars in (a), (b) and (c), (d) represent 3 µm and 500 nm, respectively.*

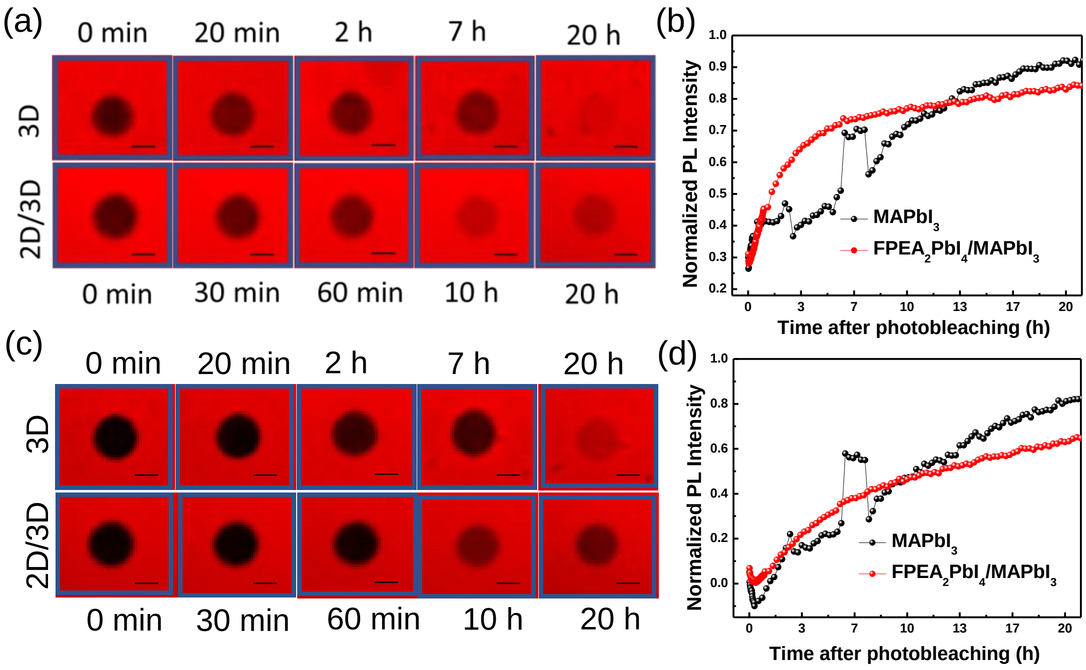

*Figure S10: Maps and plots of PL (emission >730 nm, i.e., from MAPI only), as a function of time after photodamage, of 3D MAPbI₃ and ultra-thin 2D-on-3D HaP films. (**a**) and (**c**) show the time evolution of the PL emission maps of a photodamaged region (dark circular spot). Figures (**b**) and (**d**) give PL emission intensity vs. time plots of the corresponding normalized PL intensity recovery from photodamage. 3D layers were photobleached with 488 nm laser pulses of different intensities: after the damage, the films retained ~30% (i.e., severe, ~70% photodamage; (**a, b**)) and ~0% (i.e., complete, ~100% photodamage; (**c, d**)) of their initial PL intensity. For 2D/3D bilayers, the 2D growth was terminated in regime II of the PL intensity vs. time profile, i.e., in Fig. 1c.*

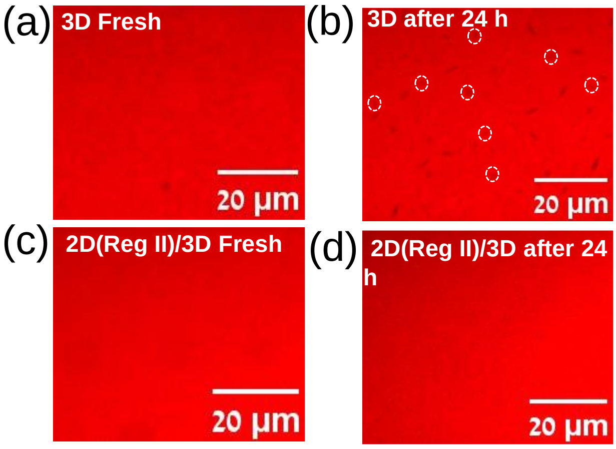

*Figure S11: Confocal images for 3D $MAPbI_3$ PL emission (>730 nm). (**a**) and (**c**) Fresh emission maps for 3D and 2D/3D HaP films, respectively. (**b**) and (**d**) maps for 3D and 2D/3D films, respectively, after 24h storage in ambient humidity (45-55% RH) and room light conditions. All films were encapsulated by a layer of poly-isobutylene. White dashed circles in (b) are used to mark some of the film's degradation spots (appear in black).*

S1. Quantitative XPS analysis and Calculation of 2D layer thicknesses

Tables S1-4 below show the experimental atomic concentration (in %) for different elemental compositions obtained at the probed locations on the samples, obtained by analyzing the F 1s, I 3d, Pb 4f, N 1s, C 1s core level spectra shown in Figs. 3 and S4. Tables S1, S2 and S3, S4 show the atomic concentrations at two different locations on each sample with 2D grown in regions II and IV of the PL intensity vs. time profile plot (cf. Fig. 1c, main text), respectively.

Table S1 (for 2D grown in Reg. II)						
Peak	Position	FWHM	Area	Atomic mass	Atomic conc.%	Sum (At. conc.%)
F 1s	687.83	1.28	8786.5	18.10	6.24	6.24
I 3d-5/2	619.60	1.11	230852.1	126.90	15.92	26.83
I 3d-3/2	631.15	1.11	158355.9	126.90	10.91	
Pb 4f-7/2	138.70	0.86	5400.5	207.21	4.76	8.44
Pb 4f-5/2	143.55	0.88	41722.3	207.21	3.68	
N 1s	402.60	1.20	5743.9	14.01	8.65	8.65
C 1s-C-Metal	284.34	0.94	700.0	12.01	1.88	49.07
C 1s-C-C	285.90	0.99	12083.9	12.01	32.50	
C 1s-C-N	286.68	0.89	2863.4	12.01	7.71	
C 1s-C-F	287.38	0.99	2591.6	12.01	6.98	

Table S2 (for 2D grown in Reg. II)						
Peak	Position	FWHM	Area	Atomic mass	Atomic conc.%	Sum (At. conc.%)
F 1s	687.63	1.31	8720.1	18.10	6.29	6.29
I 3d-5/2	619.63	1.10	219884.0	126.90	15.40	25.97
I 3d-3/2	631.08	1.09	151091.3	126.90	10.57	
Pb 4f-7/2	138.73	0.87	51709.7	207.21	4.63	8.12
Pb 4f-5/2	143.55	0.87	38958.4	207.21	3.49	
N 1s	402.45	1.12	5459.3	14.01	8.35	8.35
C 1s-C-c	285.93	0.95	13113.5	12.01	35.81	50.42
C 1s-C-N	286.68	0.91	2891.2	12.01	7.90	
C 1s-C-F	287.384	0.959	2455.5	12.01	6.71	

Table S3 (for 2D grown in Reg. IV)						
Peak	Position	FWHM	Area	Atomic mass	Atomic conc.%	Sum (At. conc.%)
F 1s	687.55	1.34	12680.9	19.00	8.19	8.19
I 3d-5/2	619.65	1.13	154712.9	126.90	9.70	16.43
I 3d-3/2	631.18	1.14	107528.3	126.90	6.73	
Pb 4f-7/2	138.75	0.86	28641.0	207.21	2.30	4.07
Pb 4f-5/2	143.58	0.87	22059.2	207.21	1.77	
N 1s	402.33	1.20	5597.8	14.01	7.66	7.66
C 1s-C-C	285.92	0.94	19400.6	12.01	47.43	63.25
C 1s-C-N	286.67	0.91	2611.8	12.01	6.39	
C 1s-C-F	287.33	0.93	3855.6	12.01	9.43	

Table S4 (for 2D grown in Reg. IV)						
Peak	Position	FWHM	Area	Atomic mass	Atomic conc.%	Sum (At. conc.%)
F 1s	687.68	1.35	15348.7	19.00	8.77	8.77
I 3d-5/2	619.60	1.14	175917.1	126.90	9.76	16.65
I 3d-3/2	631.13	1.13	124265.0	126.90	6.89	
Pb 4f-7/2	138.70	0.87	31022.1	207.21	2.20	3.86
Pb 4f-5/2	143.58	0.87	23452.6	207.21	1.66	
N 1s	402.38	1.16	6243.5	14.01	7.57	7.57
C 1s-C-C	285.92	0.92	21865.6	12.01	47.29	62.53
C 1s-C-N	286.71	0.91	3213.4	12.01	6.95	
C 1s-C-F	287.33	0.91	3830.8	12.01	8.29	

Assuming we have phase-pure $FPEA_2PbI_4$ on $MAPbI_3$/glass substrate (supported by PL, XRD, and electron diffraction experiments shown in the main text), the ideal atomic concentration ratio for different elements are:

(A) $FPEA_2PbI_4 = [FC_6H_4(CH_2)_2NH_3]_2PbI_4$

C/F=8	C/N=8	C/I=4
C/Pb=16	I/F=2	I/N=2
I/Pb=4	F/Pb=2	N/Pb=2

(B) $MAPbI_3 = CH_3NH_3PbI_3$

I/C=3	I/Pb=3	N/Pb=1
I/N=3		

According to F at. % in $FPEA_2PbI_4$ 2D layer, the contribution of the 2D layer to the total experimentally calculated atomic conc. % in Table S1 for different elements are:

N = F = 6. 24 %

Pb = F/2 = 3.12 %

C = 8F = 49.92 %

I = 2F= 12.48 %

Thickness (d) of the top 2D layer can be estimated by considering exponential attenuation of the ejected photoelectrons as a function of its (2D) thickness, assuming a uniform and homogeneous layered structure of 2D over the 3D HaP films[1]. Hence

$$d_{2D}(A^{\circ}) = \lambda \times cos\theta \times \ln\left(1 + \frac{[C_{2D}]+[N_{2D}]+[Pb_{2D}]+[I_{2D}]+[F_{2D}]}{[C_{3D}]+[N_{3D}]+[Pb_{3D}]+[I_{3D}]}\right) \qquad \text{----------------- (1)}$$

where θ is the takeoff angle with respect to the surface normal; the fraction within the bracket is the sum of photoelectron intensities due to all elements in the top 2D $FPEA_2PbI_4$ and bottom 3D $MAPbI_3$ layers, respectively. An attenuation length parameter (λ) of 25-30 A° is assumed, neglecting the kinetic energy dependencies across the measured energy range[1-3].

Thickness (d) of $FPEA_2PbI_4$, according to the F atomic concentration (I_F) (as F is the only element that is not present in the bottom $MAPbI_3$ layer), is found as follows:

$$d_{2D} = 25\text{-}30 \times 1 \times \ln\left(1 + \frac{I_F \times 12.5}{100-(I_F \times 12.5)}\right) \text{-----------------} \ (2)$$

$$= 25\text{-}30 \times 1 \times \ln\left(1 + \frac{6.24 \times 12.5}{100-(6.24 \times 12.5)}\right) \text{-----------------} \ (3)$$

$$= 37.9 - 45.2\ A°3 \approx 3.8 - 4.5\ nm\ for\ 2D\ layer\ grown\ in\ regime\ II \text{ ---------} \ (4)$$

The excess atomic concentration for different elements estimated in Table S1 is due to the contribution from the bottom $MAPbI_3$ layer, i.e.,

N = (8.65 – 6.24) % = 2.41 %

Pb = (8.44 – 3.12) % = 5.32 %

I = (26.83 – 12.48) % = 14.35 %

We do not find extra C atomic concentration that can be attributed to the bottom $MAPbI_3$ layer.

According to the above-noted values, the atomic ratios in $MAPbI_3$ are

I/Pb = 2.7

I/N = 5.95

N/Pb = 0.45

I/C = NA (See note on C atomic conc. % above and possible reasoning below).

Except for the I/Pb ratio, these values differ from the ideal stoichiometric ratios for $MAPbI_3$ perovskite. The observed deficiency of C and N species suggests that, most likely, the organic methylammonium component has escaped from the perovskite structure due to overnight pumping in the XPS instrument down to ultra-high vacuum conditions (~ 10^{-9} mbar = 10^{-7} Pa) before the measurements. Further, we note that even though the organic component of the 2D structure, $FPEA^+$, can also escape, its larger structure makes it much less likely. Even if it does, that won't affect the 2D thickness estimates as the F to N ratio in $FPEA^+$ is 1:1.

Additionally, there could be vacuum-induced degradation of 2D and 3D perovskite structures or the formation of mixed 2D-3D phases, which could have led to the observed discrepancy in the atomic conc. % of the 3D $MAPbI_3$ perovskite.

A similar analysis for estimating 2D thickness at another location of the same sample using elemental quantification data in Table 2 gives value

$$d_{2D} = 38.6 - 46.3\ A^{\circ} \approx 3.9 - 4.6\ nm$$

For Table S3, i.e., the one obtained for 2D/3D samples with a thicker 2D layer grown in regime IV of the PL intensity vs. time profile, we observe the atomic conc. % F and N are nearly the same (F/N=1.07), and no excess N can be attributed to the signal from the bottom $MAPbI_3$ (this is with the assumption that all the N signal comes from the 2D $FPEA^+$ cation. This implies that the thickness of the top $FPEA_2PbI_4$ layer is greater than the maximum x-ray probing depth, i.e., 10 nm.

Based on the elemental quantification data shown in Table S3, we find the following atomic ratios for different elements:

F/Pb = 2.01	N/Pb = 1.88	I/Pb = 4.04
I/F = 2.0	I/N = 2.14	I/N = 2.14
C/F = 7.72	C/N = 8.25	C/I = 3.85
C/Pb = 15.54		

Similar conclusions are also obtained by analyzing Table S4. All the atomic ratios are very close to their ideal stoichiometry (see (A) above) in the $FPEA_2PbI_4$ layer, which supports our claim of having a phase pure $FPEA_2PbI_4$ 2D layer on top of 3D $MAPbI_3$ perovskite. Additionally, we note that the fit of the above elemental atomic ratios contrasts with the case obtained from Tables S1 and S2 (for thin 2D-on-3D $MAPbI_3$ layers), where the deviation, esp. for C and N species, was significant. This supports our previous conclusion that the small MA^+ cations from the 3D perovskite can escape more readily under ultra-high vacuum conditions than the large $FPEA^+$ ones from the corresponding 2D HaP.

S2. UPS Analysis

The VBM positions were determined (from the low energy tail of the UPS spectrum, Fig. 4a in the main text) to be 1.3 eV and 1.6 eV below the Fermi level for the 3D and 2D/3D bilayers, respectively. For the calculation of work function Helium, I radiation energy (21.22 eV) was subtracted from the high-binding energy cut off 17.02 eV and 16.72 eV for 3D and 2D/3D bilayers (Fig. 4b in the main text), respectively.

WF_{3D} = 17.02 eV - 21.22 eV = -4.2 eV versus vacuum level ----------------- *(5)*

WF2D/3D = 16.72 eV – 21.22 eV = -4.5 eV versus vacuum level ----------------- *(6)*

The VBM versus vacuum level (i.e., the IE) was then calculated to be

VBM_{3D} = - 4.2 eV – 1.28 eV ≈ -5.5 eV

$VBM_{2D/3D}$ = - 4.5 eV – 1.63 eV ≈ -6.1 eV

S3. Confocal Photoluminescence Microscopy used for FRAP Measurements

FRAP measurements were carried out on an Olympus Fluoview confocal microscope (BX50WI). 488 nm laser excitation was used to illuminate the HaP samples and detect the resulting PL signals. The 488 nm laser was raster-scanned on the sample surfaces with a scan rate of 2.71 sec per scan. The field of view in one scan was measured in 512×512 pixels, with a laser dwell time per pixel of 7.2 μs. We used a 50x air objective with a numerical aperture of 0.75 for both exciting and collecting the PL signals from the samples. The laser power used for acquiring the PL images was measured to be 63 μW. This value, for a laser beam diameter of 0.25 μm, translates into a power density of 1.3×10^9 W/m^2 irradiated in each pulse of 7.2 μs duration on the sample. This density is equivalent to one-second illumination of the sample with energy per unit area ≈ 13×10^3 J/m^2. Comparing this value with AM 1.5 G solar illumination with irradiated energy per unit area per sec ≈10^3 Joule/m^2 (1000 W/m^2), the irradiated energy density per laser pulse is roughly equivalent to 13 suns. The localized photodamages on the HaP samples were made with 6−20 times higher laser power than those used for the PL imaging, with the same irradiation pulse duration of 7.2 μs. This is equivalent to irradiating the samples with the 80 – 250x AM 1.5 G solar irradiation energy density. The PL images were then measured at regular intervals after the photodamage to track the recovery of the PL signal in the photodamaged regions.

References

(1) Briggs, D.; Wiley, J. *Pr. Surf Anal by Auger X-ray Photoelectron Spectrosc* **1983**, *1*, 1–8. https://doi.org/10.1016/0368-2048(84)80044-4.

(2) Tanuma, S.; Powell, C. J.; Penn, D. R. Calculations of Electron Inelastic Mean Free Paths for 31 Materials. *Surf. Interface Anal.* **1988**, *11* (11), 577–589. https://doi.org/10.1002/sia.740111107.

(3) Tanuma, S.; Powell, C. J.; Penn, D. R. Calculations of Electron Inelastic Mean Free Paths for 31 Materials. *Surf. Interface Anal.* **1991**, *17* (17), 927-939.
